# Neonatal Urine Metabolic Signature Reflects Multisystemic Adaptations Linked to Preterm Birth

**DOI:** 10.3390/ijms26188953

**Published:** 2025-09-14

**Authors:** Pere Bibiloni, Jean-Charles Martin, Pilar Cobo, María Victoria Jiménez-Cabanillas, María DeLucas, Catherine Tardivel, Catalina Picó, Francisca Serra, Juana Sánchez

**Affiliations:** 1 Laboratory of Molecular Biology, Nutrition and Biotechnology (Nutrigenomics, Biomarkers and Risk Evaluation), Edifici Mateu Orfila, University of the Balearic Islands, Carretera de Valldemossa Km 7.5, 07122 Palma, Spain; pere.bibiloni@uib.eu (P.B.); maria.delucas@uib.es (M.D.); cati.pico@uib.es (C.P.); joana.sanchez@uib.es (J.S.); 2 Health Research Institute of the Balearic Islands, IdISBa, 07010 Palma, Spain; pilar.cobo@ibsalut.es (P.C.); mariavictoria.jimenezcabanillas@ssib.es (M.V.J.-C.); 3 CIBER Fisiopatología de la Obesidad y Nutrición (CIBEROBN), Instituto de Salud Carlos III (ISCIII), 28029 Madrid, Spain; 4 Centre Cardiovasculaire et Nutrition (C2VN), INRAE, INSERM, Aix Marseille Université, 13005 Marseille, France; jean-charles.martin@univ-amu.fr (J.-C.M.); c.tardivel@univ-amu.fr (C.T.); 5 Division of Neonatology, Department of Pediatrics, University Hospital Son Espases, 07010 Palma, Spain; 6 Artificial Intelligence Research Institute of the Balearic Islands (IAIB), 07122 Palma, Spain

**Keywords:** prematurity, preterm birth, metabolome, urine, LC-MS, RMN

## Abstract

Prematurity is associated with a higher risk of developing short- and long-term metabolic complications. However, the concrete mechanisms are not yet fully understood. The purpose of this study was to characterize early urinary metabolic adaptations linked to preterm birth. Urine samples collected at one month of age were compared between extremely and very preterm neonates (<32 weeks gestation) (*n* = 45) and term newborns (*n* = 96). Liquid chromatography coupled to mass spectrometry (LC-MS) and nuclear magnetic resonance (NMR) techniques were both applied and analyzed independently. Univariate analysis and multivariate analyses were utilized to assess the impact of preterm birth on the metabolites identified. Multiblock analysis was further used to evaluate the effect of prematurity on biological functions. A profound impact of prematurity was observed. Both experimental groups differed in the concentrations of 240 metabolites from the LC-MS dataset and 52 from the NMR one. Multivariate analyses confirmed a significant and important separation between groups. Finally, multiblock analysis identified six major biological outcomes affected by preterm birth: nitrogen metabolism, growth, neurochemical metabolism, microbiota metabolism, cell defense, and metabolic alterations. Most of the observed variations exhibited biological plausibility and were consistent with reported health complications associated with preterm birth. In conclusion, preterm birth is linked to a specific fingerprint in the urinary metabolome, reflecting metabolic adaptations in multiple systems occurring at one month of age.

## 1. Introduction

Preterm birth is defined as the delivery of a live infant before 37 weeks of pregnancy. It can be further categorized by gestational age as extremely preterm (<28 weeks), very preterm (between 28 and 32 weeks), or moderate/late preterm (between 32 and 37 weeks) [1]. Globally, 10% of the babies are born prematurely, with an upward trend in some particular regions. In 2020, approximately one million of neonates died worldwide because of preterm birth complications and even a greater number survived with life-lasting disabilities [2].

In the short term, prematurity is associated with multiple complications. These are diverse and involve all of the organism systems. Some of the most common ones include necrotizing enterocolitis, respiratory distress syndrome, intraventricular hemorrhage, anemia, retinopathy of prematurity, or several infections [3]. As a whole, this increases the mortality of preterm infants, to the point that preterm birth complications are the leading cause of death in children under the age of 5 years [1]. Moreover, prematurity heightens the risk of developing a wide range of pathologies in the long term: respiratory disorders, kidney diseases, neurodevelopmental disorders, cardiovascular diseases, and metabolic alterations [4]. This impact is even more significant in infants born very preterm [5] or when associated with suboptimal nutritional management [6]. The fact that a detrimental impact during early development leads to an increased risk of disorders in future stages is the core of the developmental origins of health and disease theory (DOHaD). The negative metabolic programming could be explained by direct injuries during the stressful event or by an adverse consequence of the adaptation that the organism must face to survive [7,8].

Urine is a highly suitable biofluid to study a great variety of disorders. As the primary route of excretion for polar compounds, urine provides valuable insights into systemic biochemical changes, reflecting metabolic processes occurring throughout the entire organism. Plus, it is obtained non-invasively and its analysis requires minimal sample preparation. In consequence, the application of metabolomic approaches to identify biomarkers in urine is of great interest [9]. Several studies have already focused on the impact of prematurity on the neonatal urine metabolome [10,11,12,13,14], as reviewed in [15], stating the utility of this approach for such purposes.

Despite increasing evidence highlighting the impact of prematurity on the neonatal urinary metabolome, significant gaps remain in understanding the early metabolic adaptations occurring in preterm infants, particularly in extremely and very preterm neonates. A more holistic approach—both technical and analytical—is required to deepen insights into these early metabolic changes, as they may reveal biological mechanisms that remain poorly understood.

The present article addresses the research question of which specific early urinary metabolomic changes are associated with prematurity. We hypothesized that preterm neonates exhibit a distinct urinary metabolic signature compared to term neonates, which could provide valuable insights into health outcomes linked to prematurity. To test this hypothesis, we characterized the urinary metabolic signature associated with prematurity. To this end, we designed a cross-sectional, observational, single-center study to compare the urinary metabolome at one month of age between extremely/very preterm neonates and term neonates. The novelty of this study lies in its methodological approach. We employed both liquid chromatography coupled with mass spectrometry (LC-MS) and nuclear magnetic resonance (NMR), two complementary techniques that provide broader metabolite coverage. Furthermore, data were analyzed using a multiblock approach, which integrates data at a higher biological level to enhance the interpretation of metabolic signatures. This strategy enabled the identification of a comprehensive landscape of biological functions altered by preterm birth based solely on urinary analysis, including some novel pathways such as the neurochemical metabolism.

## 2. Results

### 2.1. Demographic Characteristics of the Study Populations

#### 2.1.1. Mothers

Relevant categorical variables regarding the mothers recruited in the study are reported in Supplementary Table S1. Most of the population was composed of Caucasian women (76% in Term vs. 64% in Prem) with a minor representation of other ethnic groups. Both cohorts showed similar histories of previous pregnancies (*p* = 0.300) and breastfeeding (*p* = 0.690). On substance use (tobacco, alcohol, or drugs) during pregnancy, only exceptional cases were acknowledged without differences between groups (*p* = 0.350, *p* = 0.210, and *p* = 0.690, respectively). A strong association was found between preterm birth and multiple pregnancy (*p* < 0.001), as almost half of the prematurely born babies were twins, while only one was recruited in the Term cohort. This agrees with national data from 2010, in which approximately 54% of the preterm live births are multiple, a proportion that is even higher in very preterm births [16]. Recent data from 2023 follows the same trend [17]. Most term newborns were delivered vaginally (76%), whereas cesarean section was the predominant mode of delivery for preterm births (67%, *p* < 0.001) which is in accordance with current national records [17,18]. Additionally, preterm mothers had a lower breastfeeding success rate compared to full-term mothers (*p* < 0.001), in agreement with previous figures from a recent national study [19].

Concerning anthropometric characteristics of the recruited mothers (Supplementary Table S2), volunteers from both cohorts had a median age of 36 years (*p* = 0.780). There were no significant differences between groups in terms of body mass index (BMI), either before pregnancy (*p* = 0.539) or one month after delivery (*p* = 0.411). In accordance with the study design, gestational age between groups differed by almost 10 weeks (*p* < 0.001). Mothers who gave birth prematurely presented a lower body fat percentage (BFP, *p* = 0.007) and a higher proportion of muscle mass (MMP, *p* < 0.001) compared to mothers of term babies. These differences were expected, considering that 95% of the total gestational weight gain occurs during the second and third trimesters of pregnancy. Besides the products of conception, the majority of this weight gain is attributed to fat deposits [20]. The increase in adiposity can be mainly attributed to subcutaneous fat accumulation, as no differences in visceral fat were found (*p* = 0.408), which also agrees with previous knowledge [20].

#### 2.1.2. Neonates

Infants’ anthropometric features are summarized in Supplementary Table S3. At birth, preterm neonates had lower body weight, body length, and head circumference compared to term newborns (*p* < 0.001 in all cases). When expressed as percentiles corrected for gestational age, differences in weight and length remained significant (*p* = 0.001 and *p* < 0.001, respectively). At one month of age, preterm babies presented lower values for all features measured, both in absolute terms and corrected for gestational age (*p* < 0.001 in all cases). Finally, concerning the growth rate during the first month of life, preterm infants presented a lower body weight gain compared to term infants (*p* < 0.001), while no differences between length gain (*p* = 0.189) and head circumference increment (*p* = 0.755) were seen. On the other hand, when correcting for gestational age, in the preterm babies an overall trend of diminishing the percentile was found for all three features (*p* < 0.001 in all cases). This suggested a compromised early growth in comparison to the Term group, which showed discrete increases in the percentiles.

### 2.2. Urine Metabolomics

#### 2.2.1. Univariate Analysis (UVA) of Metabolites Identified

**Figure 1:**
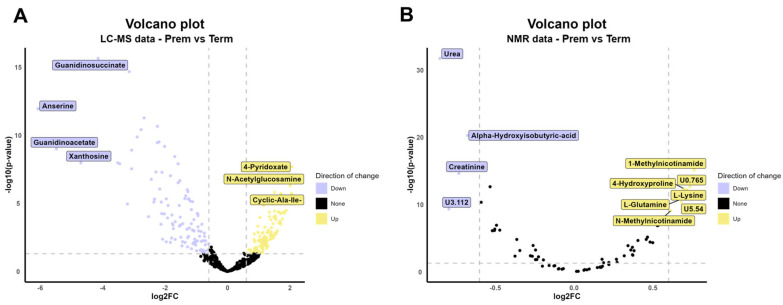
Volcano plot displayed for LC-MS data (**A**) and NMR data (**B**). FC calculated with Term group as reference. FDR threshold stablished at <0.05 and |log_2_FC| > 0.6. Plots constructed with R package ggplot2 v3.5.1 [21] and ggrepel v0.9.6 [22].

The identified differences between the preterm and full-term groups at the level of urinary metabolome were explored in detail. Data from the two techniques applied (LC-MS and NMR) were analyzed independently, and all data are included in Supplementary Table S4. LC-MS enabled the identification and semi-quantification of 521 compounds, while NMR allowed the annotation of 74 ROI (62 metabolites and 12 unknown regions). Of these, 38 metabolites were common in both techniques. Both analytical approaches showed consistent directional changes in most metabolite levels. Regarding LC-MS dataset, a total of 122 metabolites displayed higher concentrations in preterm neonates compared to term newborns, while 118 had lower levels. From NMR results, 28 showed increased levels while 24 were reduced (Supplementary data). Volcano plot analysis contributed to reveal that, although the number of significantly changed metabolites was relatively balanced in both techniques, the magnitude of change was greater among the down-regulated in the LC-MS dataset compared with the NMR (Figure 1).

Metabolites with lower levels in premature babies compared to term newborns involved compounds deeply linked to the renal function, such as guanidinosuccinate, guanidinoacetate, urea or creatinine; the dipeptide anserine, the nucleoside xanthosine or the organic acid alpha-hydroxyisobutyric. On the opposite, metabolites that displayed higher concentrations in preterm neonates in relation to term newborns included vitamin derivates, like 4-pyridoxate, 1-methylnicotinamide or N-methylnicotinamide; amino acid related metabolites, such as cyclic Ala-Ile, L-lysine, L-glutamine or 4-hydroxyproline; or N-acetylglucosamine, a component of glycoproteins and glycosaminoglycans (Supplementary Table S5).

#### 2.2.2. Multivariate Analysis (MVA) of Urinary Metabolome

**Figure 2:**
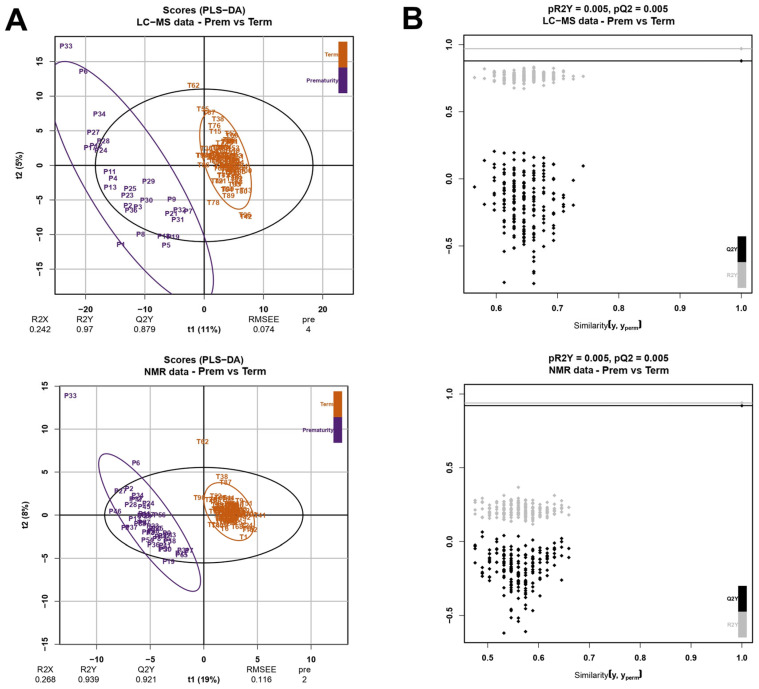
(**A**) PLS-DA 2D score plots for LC-MS (top) and NMR (bottom) showing the distribution of Prem and Term samples according to components 1 and 2. Metrics evaluating the performance of the model (R2Y, Q2Y) are shown. Plots constructed with R package ropls v1.32.0 [23]. (**B**) Permutation plot of PLS-DA models created from LC-MS and NMR data (top and bottom, respectively). Number of permutations = 200. Plots constructed with R package ropls v1.32.0 [23].

UVA provided an initial overview of urinary metabolites affected by prematurity. However, this data analysis only offered a narrow perspective on metabolic changes. To comprehensively assess the global impact of prematurity on the neonatal metabolome in a holistic manner, MVA models were constructed. A remarkable metabolic signature was detected in the premature group, as PLS-DA showed a clear distinction between groups either with LC-MS or NMR data (Figure 2A). Preterm samples exhibited greater metabolic variability compared to Term specimens, which showed a more homogeneous metabolome. This could be attributed to the inherent heterogeneity of this phenotype, arising from diverse etiologies of preterm birth and the individual developmental trajectories of each premature infant. The models displayed exceptional performance, both for their goodness of fit and their predictive ability (estimated by the R2Y and the Q2Y metrics, respectively), with values close to 0.9. A permutation test confirmed the statistical significance of these, as both pR2Y and pQ2 were lower than the significance threshold (Figure 2B).

**Figure 3:**
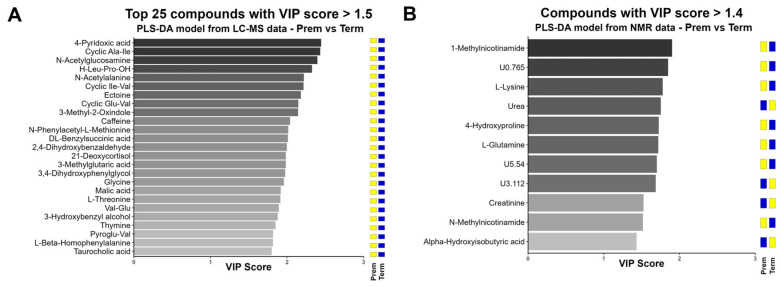
VIP scores plot for the top 25 compounds with VIP score higher than the threshold (set at 1.5) from the PLS-DA model from LC-MS data (**A**) and all compounds with VIP score higher than the threshold (set at 1.4) from the PLS-DA model from NMR data (**B**). The tiles at the right indicate the sense of the variation from the log_2_FC: yellow for positive, blue for negative (Term group as reference). Plots constructed with R package ggplot2 v3.5.1 [21]. For the complete set of metabolites from LC-MS data, please see Supplementary Figure S2.

Variable importance projection (VIP) scores were calculated for the compounds introduced in the PLS-DA models. The significant threshold for VIP scores was determined using a normal probability plot, identifying the VIP value at which metabolites deviated the most from normal distribution (Supplementary Figure S1). Thresholds were delimited to 1.5 for LC-MS data and 1.4 for NMR data, leading to the selection of 62 and 11 compounds, respectively (Figure 3 and Supplementary Figure S2).

The top discriminant metabolites from the LC-MS data comprised compounds detected by UVA, in addition to other metabolically related compounds. The top 25 included the vitamin derivate 4-pyridoxic acid, followed by the dipeptides cyclic Ala-Ile, Leu-Pro, cyclic Ile-Val, cyclic Glu-Val, Val-Glu and pyroGlu-Val; N-acetylglucosamine, which appeared in third position; as well as other amino acids (L-Thr, Gly) or modified amino acids (N-acetylAla, N-Phenylacetyl-L-Met, L-beta-homoPhe). All these compounds showed higher concentrations in the Prem group. As observed with LC-MS data, the discriminant compounds from NMR data included metabolites that exhibited elevated levels in the Prem group, such as the vitamin metabolite 1-methylnicotinamide (the most significant) and the amino acids L-Lys and 4-Hyp. However, this analysis was also sensitive to compounds which showed lower levels in preterm neonates, such as urea and creatinine, both relevant markers of renal function.

Creatinine, L-lysine, 4-hydroxyproline, glycine, and oxoglutaric acid appeared as relevant VIPs in both approaches (LC-MS and NMR) and showed the same direction of change. L-glutamine and xanthosine displayed discrepancies between methods, which discarded these compounds as good discriminants between both groups.

Additionally, subgroup analyses were performed to disentangle the effects of prematurity from those of potential confounding factors. Specifically, MVA models were created within both the Prem and Term groups, stratified according to the variables: infant sex, feeding patterns, delivery mode, type of pregnancy (single or multiple), and the presence or absence of gestational diseases (Supplementary Figure S3). Although these factors introduced some variability within each group, the differences between Prem and Term neonates remained evident, and no significant overlap was observed within any subgroup. This suggests that these factors neither hindered nor amplified the effect attributed to prematurity. Therefore, the influence of these confounding variables appears to be limited, and the results discussed are most likely attributable to the preterm birth phenotype.

#### 2.2.3. Multiblock Analysis of Urinary Metabolome

To facilitate data interpretation within a physiological context, variables were organized into functional blocks based on their biological roles. As a result, 66 modules for the LC-MS approach and 14 for the NMR technique were built (Supplementary Table S5). In order to ensure that this procedure did not distort the distribution of observations, the score plots of original data and weighted blocked data were compared (Supplementary Figure S4). In both cases, a similar mapping was obtained and the models remained statistically significant.

**Figure 4:**
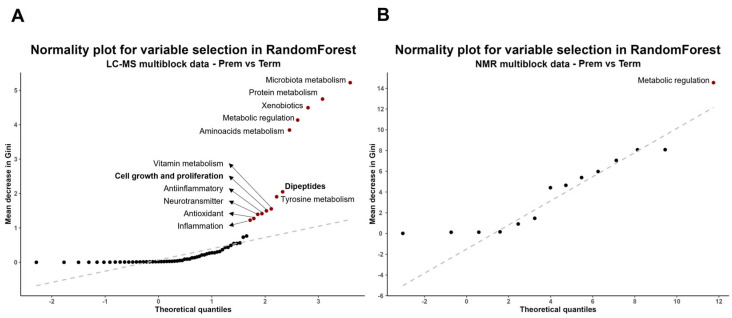
Biological functions identified by the multiblock analysis as altered in Prem vs. Term contrast. Normality plot for the VIP scores (Mean Decreased Gini values) obtained in a random forest analysis from LC-MS data (**A**) and from NMR data (**B**). Points in red and labeled are considered the most interesting as they have higher VIP scores and deviate from normal distribution. Plot constructed with R package ggplot2 v3.5.1 and qqplotr v0.0.6 [21,24].

The impact of prematurity on biological functions was initially assessed by identifying the most dysregulated blocks between groups. Random forest analysis was applied to that purpose, leading to the selection of eleven blocks from LC-MS data and a single one from NMR data (Figure 4). These included, in order of decreasing importance: microbiota metabolism, protein metabolism, xenobiotics, metabolic regulation (from both analytical approaches), amino acids metabolism, dipeptides, tyrosine metabolism, vitamin metabolism, cell growth and proliferation, anti-inflammatory, neurotransmitters, antioxidants and inflammation.

**Figure 5:**
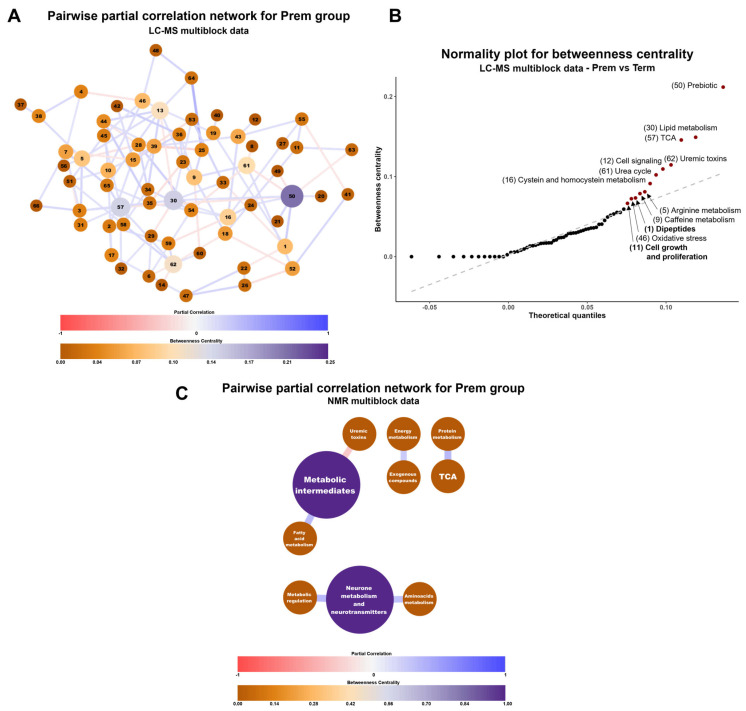
Pairwise partial correlation networks for multiblock analysis from LC-MS data (**A**) and NMR data (**C**). For the legend of LC-MS blocks, please see Supplementary Figure S5. Only interactions specific to the Prem group are shown. The size and color of the nodes indicate their betweenness centrality degree (orange and small the lowest; purple and big the highest). The size and boldness of the edges represent the strength of the correlation (the wider and bolder, the stronger). Red edges correspond to negative correlations, blue edges correspond to positive correlations. Plots constructed with Cytoscape v3.10.1 [25]. (**B**) shows the normality plot of the betweenness centrality scores for the biological functions from LC-MS multiblock data. Points in red and labeled are considered the most interesting as they have higher scores and deviate from normal distribution. Plot constructed with R package ggplot2 v3.5.1 and qqplotr v0.0.6 [21,24].

Subsequently, the most influential functions in the preterm biological system were determined using a pairwise partial correlation network analysis (Figure 5A). This approach allowed the identification of pathways with substantial impact on the regulation of the whole biological system, as quantified by their betweenness centrality degree. Specifically, twelve pathways from LC-MS data (Figure 5B), namely prebiotics, lipid metabolism, tricarboxylic acid cycle (TCA), uremic toxins, cell signaling, urea cycle, cysteine and homocysteine metabolism, arginine metabolism, caffeine metabolism, dipeptides, oxidative stress and, cell growth and proliferation were identified. From NMR data, two routes were highlighted (Figure 5C): neuronal metabolism and neurotransmitters and a metabolic intermediates category, comprising several different compounds that are key intermediaries in multiple cellular pathways.

**Figure 6:**
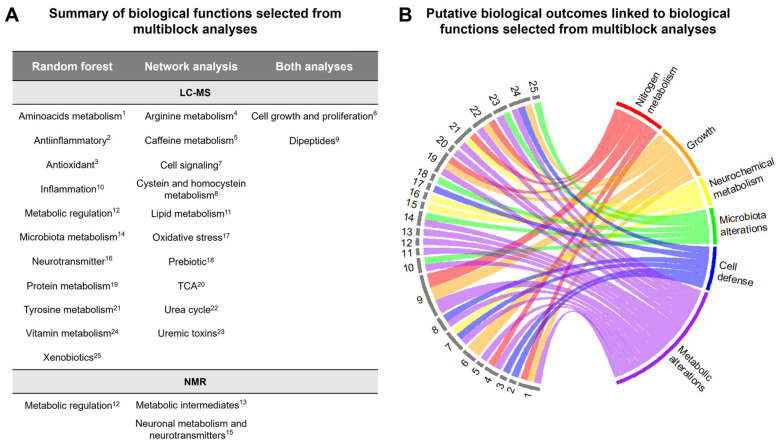
Summary of the multiblock analysis results. (**A**) Biological functions identified as most deregulated in Prem compared to Term (from random forest analysis), most impacting in the Prem biological system (from pairwise partial correlation network analysis) and from both analyses. Both approaches are shown. (**B**) Cordplot linking the biological functions (left, legend) in (**A**) to putative phenotypic outcomes (right). Plot constructed with R package circlize v0.4.16 [26].

A summary of both outcomes from multiblock analyses is presented in Figure 6A. Through this dual analysis, two key functions were common from both approaches—dipeptides and cell growth/proliferation. As a whole, LC-MS retrieved twenty-three blocks of interest, while the NMR data analysis resulted in the identification of three significant functions. To gain a comprehensive perspective on the impact of prematurity and facilitate the interpretation of its global effects on metabolism, all functional blocks were mapped to putative outcomes at a higher biological level (Figure 6B). This highlighted a urinary metabolome signature for preterm neonates involving adaptations in biological pathways related with nitrogen metabolism, growth, neurochemical metabolism, microbiota alterations, cell defense and metabolic alterations.

## 3. Discussion

Prematurity is a stressful event for the newborn, with short-term multisystemic consequences and adaptations that can even persist into adulthood [3]. In this context, the differences in the urine metabolome between (very and extremely) preterm and full-term neonates at one month of age have been studied, aiming to better understand the metabolic signature and underlying mechanisms of prematurity. Urine was selected for its non-invasive nature and ability to reflect global metabolic shifts [9]. In order to analyze the complex urine composition, LC-MS and NMR were employed, which permitted to broaden the coverage of detectable metabolites and offered consistent outcomes. Results showed an important metabolic fingerprint of preterm birth on the urinary composition at one month of age. Overall, almost 50% of the compounds identified showed differences between groups, and a considerable number of metabolites presented a log_2_FC > 0.6 (variation over 50%), stating an effect of great magnitude. The multiblock analysis revealed a significant impact of preterm birth on several biological outcomes: nitrogen metabolism, growth, neurochemical metabolism, microbiota metabolism, cell defense, and metabolic alterations (Figure 6). The biological plausibility of these changes will be discussed next individually.

Premature newborns displayed a specific metabolome signature related to nitrogen metabolism at different levels: the metabolism of particular amino acids as arginine and tyrosine, the metabolism of proteins, its catabolism (as dipeptides are generated), and the urea cycle. Previous studies describe this general effect in preterm infants, particularly at pathways related to amino acids biosynthesis, arginine and tyrosine metabolism, and urea cycle [10]. The observed major excretion of amino acids in preterm infants is generally accompanied by lower plasma levels, potentially explained by a still-immature reabsorption mechanism in the kidney [27]. Such increased urinary loss of amino acids, plus the lower levels of urea displayed in preterm infants, could imply a suboptimal handling of protein turnover and hepatic urea synthesis. Indeed, preclinical studies in piglets suggest that preterm birth blunts the protein synthesis in the skeletal muscle in response to hormonal and nutrient signals [28,29]. Moreover, human studies observe a higher oxidation of proteins and a reduced suppression of proteolysis after an infusion of amino acids in preterm babies compared to full-term infants [30]. Altogether, these metabolic adaptations observed could explain the lower lean mass and higher fat mass found in preterm neonates compared to term newborns [31], which might be associated with an increased risk of adiposity and insulin resistance observed in adult stages [32]. Interestingly, this metabolic signature is still maintained in the urinary metabolome of young adults born prematurely, such as the impact on arginine and tyrosine metabolism, as well as on the urea cycle [33,34]; supporting the long-lasting repercussions of preterm birth.

Interestingly, a metabolic fingerprint associated with “growth” itself was also found in the Prem group. This encompassed the above described changes at protein level, along with variations in metabolites involved in cell growth and proliferation, and vitamin-related compounds. Concretely, hydroxyproline, which reflects collagen metabolism and is considered an indicator of infant development, was exacerbated in the Prem group compared to Term group, which agrees with previous findings [35]. Vitamin levels are crucial to ensure normal neonatal growth. In the Prem group, we found increased levels of metabolites related to vitamin B2 (Riboflavin), vitamin B3 (1-Methylnicotinamide and N-Methylnicotinamide), vitamin B6 (4-Pyridoxate), vitamin B7 (Biotin; Pimelic acid) and vitamin E (Delta-Cehc); as well as decreased levels of methylmalonic acid, suggesting higher vitamin B12 levels. This profile could be associated with formula feeding, more common in the Prem group, which often includes higher-than-required vitamin supplementation to ensure adequacy [36]. Interestingly, excess B vitamin intake has been linked to increased body fat gain and a higher risk of obesity [37], complications associated with preterm birth and formula feeding. At the clinical level, our somatometric measurements also reflected an affected neonatal growth. Premature infants were smaller than their term counterparts both at birth and at one month of age, with a slower growth rate reflected in their declining percentile evolution. Slow early growth in preterm infants is normal [31] and typically followed by a catch-up period. An adequate process is essential, but a rapid catch-up has been linked to an increased risk of metabolic syndrome in adulthood, and its failure may increase the risk of neurological deficits [38].

In line with this evidence, our results indicated an effect of preterm birth on metabolites related to neurochemical metabolism, suggesting involvement of neuronal metabolism, neurotransmitters, cell signaling, and tyrosine metabolism. Concretely, preterm infants displayed lower levels of several neurotransmitters (γ-aminobutyric acid, serotonin, taurine), despite higher concentrations of certain precursors (Tyr, γ-Hydroxybutyric acid) and derivates (3,4-Dihydroxyphenylglycol), suggesting shifts in the neurotransmitter metabolism pathways. The Prem group also displayed lower levels of inosine, which is known for its neuroprotective role [39]. To our best extent, this is the first time the urinary analysis of preterm infants reveals alterations in the neurotransmitter metabolism compared to term neonates. This metabolic profile is in accordance with altered development of cortical areas, changes in subcortical regions as well as abnormalities in white matter observed in preterm infants, which correlates with poorer neurodevelopment [40]. In the long term, children born very prematurely show cognitive deficits and atypical psychosocial conducts [5]. Growing evidence supports the influence of perinatal factors on the onset of neurodevelopmental disorders [41]. Recently, it has been suggested that an intestinal dysbiosis could be involved in this neurodevelopmental vulnerability through the microbiota–gut–brain axis [42].

This latter hypothesis would agree with the disturbance observed on the microbiota metabolism in the Prem group. At a more particular level, lower levels of dimethylamine and 1-methylhistidine and higher concentrations of N-methylnicotinamide have been observed, variations previously attributed to microbiota changes as microbiota critically regulate the production and metabolism of methylated amines and histidine-derived compounds [11,14,43,44]. It is known that microbiome assembly in premature infants significantly differs from that of term neonates [45,46], likely due to controlled environments, antibiotics exposure, histamine H2-receptor antagonist blockers, and dietary interventions [47]. In fact, the increase in N-methylnicotinamide herein observed has been proposed as a biomarker of antibiotic suppression of the intestinal microbial community [48]. Other contributing factors to these microbiota disturbances may be formula feeding [49,50] and C-section [51], both of them more frequent in the Prem group. Microbiota of preterm infants may play a role in the onset of several health complications, such as neonatal necrotizing enterocolitis, early respiratory infections, inflammation, or even poor neurodevelopment [47]. The sustained metabolic divergences—particularly in microbial co-metabolism pathways—suggest systemic dysregulation spanning both host physiology and microbial ecology. Critically, these multi-omic signatures persist beyond postnatal developmental windows [33], implicating preterm birth as a modifier of long-term diet-microbe-host axis interactions.

Cell defense appeared as another key aspect of the prematurity phenotype. Specific metabolite variations suggested increased oxidative stress, such as higher ophthalmate, a marker of hepatic glutathione depletion [52]; and lower N-phenylacetylglycine levels, which correlates with glutathione antioxidant capacity [53]. On the other hand, compounds related to cell responses aiming to cope with this disbalance arose as significantly different in the Prem group—antiinflammation, cell signaling, or vitamins. Among these, premature newborns displayed higher salicylamide levels, an anti-inflammatory drug previously proposed as a maternal biomarker of risk for preterm delivery [54]. In preterm infants, oxidative stress likely results from the abrupt transition to a rich oxygen environment and a relative antioxidant deficiency [55]. Additionally, their immature immune system limits cytokine production, contributing to a heightened inflammatory state [56]. Altogether, this panorama is linked to the onset of several diseases such as intraventricular hemorrhage, retinopathy of prematurity, bronchopulmonary dysplasia, necrotizing enterocolitis, early-onset sepsis or bacterial infections [55,56]. Moreover, oxidative stress has been postulated as a possible mechanism explaining the onset of cardiovascular diseases later in adulthood [57]. Indeed, indoxyl sulfate, a microbiota-derived marker of oxidative stress, also displayed higher concentrations in preterm neonates compared to term newborns, similarly to previous studies [11] and has been postulated as a cardiotoxin [58].

Finally, a broad metabolic impact was revealed in the prematurity phenotype. In addition to metabolites linked with the biological functions discussed above, variations in metabolic intermediaries and related to the tricarboxylic acid cycle (TCA), lipid metabolism, uremic toxins, and multiple metabolic disorders appeared as discriminant between the groups. Regarding TCA, the organic acids cis-aconitic, oxoglutaric, fumaric and malic shown elevated levels, consistent with prior findings suggesting an enhancement of TCA in preterm babies, probably due to their high metabolic turnover [11,35]. Concerning lipid metabolism, lower levels of acylcarnitines (nonanoylcarnitine, trans-2-decenoylcarnitine) suggested enhanced mitochondrial fatty acid β-oxidation [59]; while increased 3-hydroxybutyric acid indicated ketogenesis. These results align with an enhanced usage of fatty acids as energy fuel in prematurity [60] and general adaptations on lipid metabolism [11]. Tyrosine and tryptophan, two amino acids with important roles as precursors in several routes of metabolism, showed a relevant role in the prematurity phenotype, in agreement with the available literature [10,11]. In reference to uremic toxins, preterm infants exhibited higher levels of N-acetylalanine, indoxyl sulfate and p-Cresol sulfate compared to full-term newborns, all considered uremic toxins despite their diverse origins [61]. Their retention, linked to renal dysfunction, may harm other tissues and organs, agreeing with systemic effects previously discussed. This renal disturbance is further supported by the limited excretion of creatinine, attributable to the immaturity of the renal tubules as noted in previous studies [11], as well as by reduced guanidinacetate levels, a creatine precursor whose production declines with renal malfunction [62]. In addition, chemical compounds associated with metabolic disorders were also relevant in the comparison of Prem vs. Term. The increase in some of those is linked to inborn errors of metabolism, such as N-acetylalanine and 3-methylglutaric acid, which correspond to aminoacylase I deficiency [63] and 3-hydroxy-3-methylglutaryl CoA lyase deficiency [64], respectively. Although a genetic origin is not probable, this could reflect a general metabolic disturbance resembling these phenotypes. As a whole, these fluctuations involving key metabolic pathways could hypothetically have long-term health implications for preterm infants. Changes in energy metabolism may play a role in the heightened risk of obesity and associated metabolic diseases in preterm children, such as type 2 diabetes mellitus or lipid disorders [4]. In addition, the altered renal function could also reflect the interruption of fetal nephrogenesis, potentially predisposing to future kidney diseases [65].

Our study has several strengths. Firstly, it employed a robust methodological approach, combining two complementary metabolomic techniques (LC-MS and NMR). This approach expanded metabolite coverage and increased the robustness of our findings, as both methodologies yielded consistent results. Secondly, the multiblock analytical approach allowed us to effectively link metabolite changes to higher-level biological functions. Altogether, this holistic strategy provided a comprehensive view of the metabolic signature, which, in turn, enabled the identification of pathways not previously reported as associated with preterm birth, such as neurotransmitter biosynthesis (to the best of our knowledge). Nonetheless, several limitations that affect the generalizability of the present study must be acknowledged. First, cohort heterogeneity inherent to the prematurity, like differences in feeding patterns, twin proportion, delivery mode, or gestational complications between experimental groups, could act as potential confounders. Although these variables were accounted for in specific statistical analyses and their impact appeared limited, their biological implications should not be overlooked. Second, we cannot rule out the effect of some other variables that were not specifically recorded (e.g., neonatal complications, newborn screening results, time since last feeding, which could affect circadian rhythms). Finally, the cross-sectional design of the study precludes the extrapolation of our findings to future health complications in premature infants. While our results are supported by the existing literature, longitudinal studies are required to further establish such associations.

## 4. Materials and Methods

**Figure 7:**
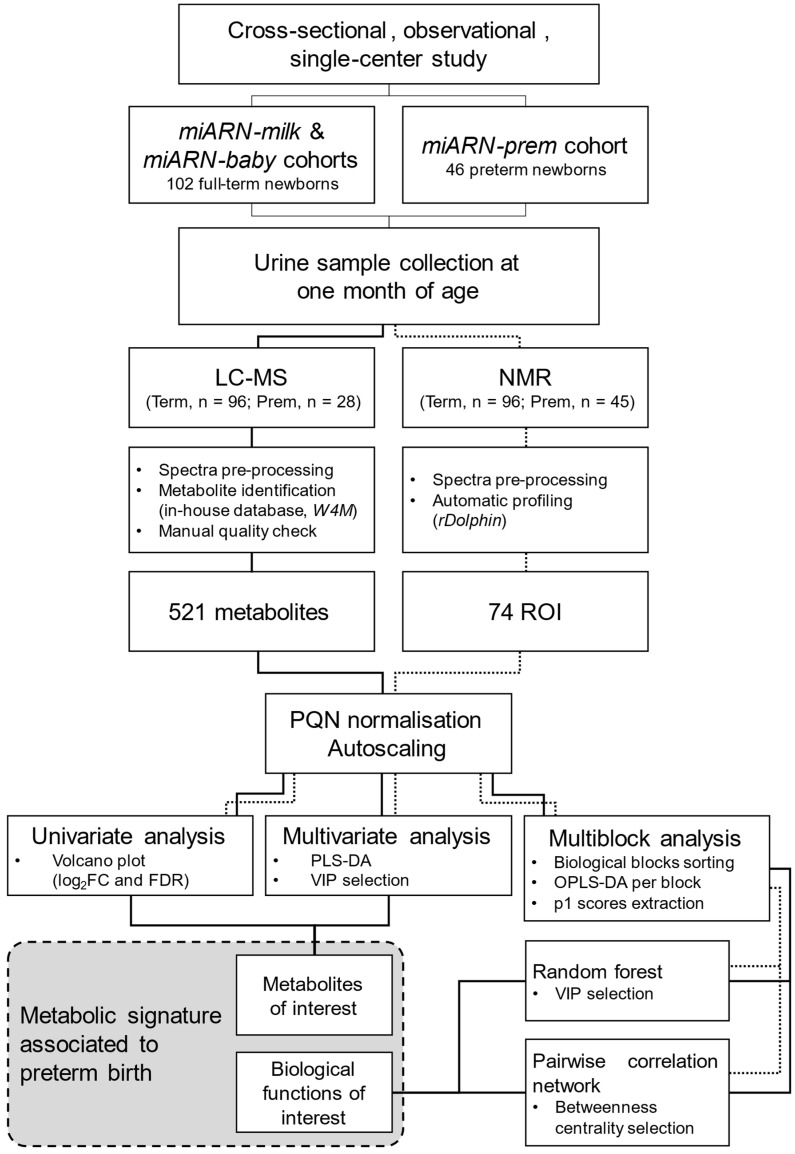
Study flow chart. LC-MS, liquid chromatography coupled to mass spectrometry; NMR, nuclear magnetic resonance; Term, full-term newborns group; Prem, preterm newborns group; W4M, workflow4metabolomics; ROI, regions of interest; PQN, probabilistic quotient normalization; FC, fold change; FDR, fold discovery rate; PLS-DA, Partial Least Squares Discriminant Analysis; VIP, Variable Importance in Projection; OPLS-DA; orthogonal PLS-DA.

This study was a cross-sectional, observational, single-center study designed to compare the metabolic profiles of preterm and term neonates using LC-MS and NMR techniques. A summary of the study design, experimental procedure and data analysis is depicted in the flow chart of Figure 7.

### 4.1. Subjects

Participants included in the current study were enrolled from two different cohorts: miARN-milk/miARN-baby and miARN-prem (approved by the Ethics Committee for Research of Balearic Islands with the codes CEI-IB IB3464/17 PI, CEI-IB 3716/18 PI, and CEI-IB 4412/20 PI, respectively). Women were approached in the neonatal unit of the reference hospital of the Autonomous Community of Balearic Islands (Hospital Universitari Son Espases) and provided written informed consent for both themselves and their babies.

miARN-milk/miARN-baby studies recruited 102 baby-mother pairs at birth, born at term (around week 40 of pregnancy, Term group) from July 2018 to March 2020. Inclusion criteria for the mothers were: (1) age between 18 and 45 years old; (2) absence of known diseases; (3) no recent changes in the regular diet (as self-reported by volunteers); (4) no drug consumption (besides nutritional supplements); (5) no history of drugs or alcohol abuse; and (6) with the intention of exclusive breastfeeding.

miARN-prem involved 46 baby-mother pairs also recruited at birth between July 2021 and March 2023. In this case, only infants born extremely prematurely or very prematurely (before week 32 of gestation) or under 1500 g of weight were included (Prem group). Inclusion criteria were considered the same (1)–(5) from the former study.

In both cohorts, mothers did not have any notable diseases prior to pregnancy (e.g., cancer). Gestational diseases were not exclusion criteria; however, they were recorded and accounted for as confounding factors. The use of in vitro fertilization assistance was not considered as a factor during recruitment and was not recorded. At the time of urine sampling, neonatal complications or results of neonatal screening were not collected.

### 4.2. Sample Acquisition and Anthropometrical Measurements

Urine samples were collected from both term and preterm infants at one month of age using “Temapar” sterile pediatric urine collection bags (Temaer Hospitalaria SA, Madrid, Spain). The one-month time point was chosen because it represents an early stage of postnatal life where metabolic adaptations linked to prematurity can still be detected, while also allowing for the relative stabilization of preterm neonates. This timing additionally coincides with a standard pediatric visit, facilitating sampling in a consistent and stress-free manner. All samples were collected between 9:00 a.m. and 2:30 p.m. Time since last feeding was not controlled, as infants were fed on demand. Immediately after specimen recovery, urine was transferred to sterile cryovials and stored at −80 °C until analyses were performed. Samples were not centrifuged to remove cells and debris prior to freezing and storage because of technical limitations at the time of collection. In the present study, 96 urine samples from the full-term cohort and 45 from the premature cohort were analyzed.

Concerning neonates, somatometric parameters (body weight, body length, and head circumference) were measured. To assess the infant growth rate, the percentile for each value was calculated, adjusted to gestational age and sex, and according to the current national standards [66,67].

Regarding the mothers, anthropometric measurements were taken on the same day as the babies. These included weight, height, and body composition. Pre-gestational data was also obtained from clinical records and self-reported questionnaires.

### 4.3. LC-MS Analysis

Metabolomic analyses using LC-MS were performed on the Biomet platform at Aix-Marseille Université (DIONEX UltiMate 3000 HPLC & ESI-Q Exactive Plus mass spectrometer, Thermo Fisher Scientific, Villebon sur Yvette, France). A total of 128 urine samples (96 from term babies and 28 from preterm babies) were prepared for the analysis. This technique was applied before the end of recruitment; therefore, only a subset of preterm neonates was included. Specimens were first thawed and centrifuged at 11,000 rpm for 15 min at 4 °C. 350 μL of the supernatant was filtered (0.45 μm) and 50 μL was transferred to a vial for analysis. Simultaneously, a pool of all samples (quality control, QC) and blanks with distilled water were arranged.

Chromatography was conducted on two distinct columns (C18 [Thermo Fisher Scientific] and HILIC [Merck Millipore, Billerica, MA, USA]) and in both positive and negative ionization for each chromatographic condition in order to extend the coverage of metabolites. The workflow followed for all samples was the same. On the C18 column, samples were analyzed during a 16 min gradient at 40 °C with a solvent rate of 0.400 mL/min (solvent A: 0.1% formic acid [Thermo Fisher Scientific] in H_2_O and solvent B: 0.1% formic acid in acetonitrile [Merck, Molsheim, France]). For the HILIC column, solvents A and B were applied (16 mM ammonium formate in H_2_O and 0.1% formic acid in acetonitrile, respectively) at a rate of 0.250 mL/min, during a 27 min gradient at 25 °C. A QC sample was injected every five samples to monitor and correct for instrumental drift and ensure consistent performance throughout the analytical sequence. Blanks were injected at the beginning of the batch. Additionally, tandem mass spectrometry (MS/MS) experiments were conducted on the QC samples at the end of the sequence. These permitted to improve annotation by comparing with the MS/MS spectra collected in the comprehensive in-house database, which contains spectral information for over 1300 compounds.

Data was extracted by the MzMine 3 software version 3.2.0 [68] from raw data acquired in FullScan. The four resulting matrices (C18 and HILIC, positive and negative) were merged. Processing of the spectra was next performed. Briefly, the workflow consisted of removing chemical noise, eliminating poorly integrated peaks, clustering isotopic signals, aligning chromatographic features across samples, and subtracting signals present in blank samples. Finally, signals with a coefficient of variation greater than 30 among the QC samples were discarded. The resulting variables were matched with the internal database containing over 1000 pure metabolites with their mass spectra and retention time. The data in-house LCMS tool of the W4M platform was used for that purpose (Galaxy software version 22.05) [69]. As a last step, automatic annotation was manually checked considering peak quality, MS/MS fragmentation patterns, and correlations between signals of the same compound. When the same metabolite was identified in both ionization modes and/or both chromatographic conditions, the one with the least CV in QC and with the highest intensity in either analytical mode was retained. In the end, 521 metabolites were identified and annotated.

### 4.4. NMR Analysis

^1^H-NMR was applied with the instrumentation available at the University of the Balearic Islands. One hundred forty-one samples were analyzed (96 from term babies and 45 from premature). To prepare samples, urine was thawed and 350 µL was mixed with 150 µL of distilled water. 50 µL of phosphate buffer (pH = 7.4, 1.5 M [reagents from Panreac, Barcelona, Spain], prepared in D2O [Eurisotop, Gif-sur-Yvette, France]) containing tetramethylsilylpropanoic acid (TSP, Eurisotop) at a final concentration of 0.8 mM was added. The mixture was centrifuged at 13.400 g for 5 min and the supernatant was transferred to a 5 mm NMR tube (Eurisotop). Simultaneously, blank samples, including only buffer and water, were prepared and processed with the same protocol.

Spectra were acquired at 600.10 MHz on a Bruker Avance III 600 spectrometer (Bruker, Billerica, MA, USA) at 298 K. For all samples, a NOESY pulse sequence was employed, collecting a total of 64 scans with a spectral width of 12,335.53 Hz and a 90° pulse (11.4 µs). The acquisition time was 2.65 s, followed by a 4 s relaxation delay with water signal suppression. To aid in metabolite structural elucidation, 2D spectra were acquired under identical temperature and frequency conditions in a subset of samples. Specifically, a DIPSI pulse sequence was used to collect 48 scans with spectral widths of 7211.54 Hz (F1) and 7201.23 Hz (F2). Acquisition times were 0.14 s (F1) and 0.03 s (F2).

Both 1D and 2D spectra were manually phased and calibrated, considering the chemical shift of TSP as a reference at 0.000 ppm. Next, automatic profiling was performed using the open-source rDolphin tool v0.2.0 [70]. Spectra were imported with a bucket resolution of 0.0006 ppm and suppressing the region from 5.1 to 4.7 ppm, corresponding to the water signal. Data available from the open Human Metabolome Database (HMDB) Version 5.0 [71] was used to annotate metabolites. Parameters for peak identification and quantification (chemical shift, tolerance, multiplicity, J coupling, bandwidth) were manually adjusted for optimal fit in each specific region of interest (ROI). Signals corresponding to the same compound were summarized as the mean if their areas were highly correlated and their peaks properly quantified. If not the case, the best signal was chosen. Signals appearing in the blanks were systematically suppressed. In the end, 74 ROIs were identified and integrated. Absolute concentrations were calculated considering the internal standard peak area.

### 4.5. Data Analysis

For all statistical analyses, R version 4.3.1 was the software utilized [72].

Clinical features were compared between groups of study. For categorical variables, Fisher’s exact test was applied to assess statistical significance, and the effect size was evaluated using Cramer’s V statistic. For numeric features, the Lilliefors test was applied to assess normality of distribution, and either the Mann–Whitney U test or *t*-test was utilized according to the previous result. The effect size for numeric comparisons was evaluated using the Cohen’s d statistic.

Regarding the metabolome dataset, probabilistic quotient normalization (PQN) was applied as the first step. This method aims to normalize data and minimize the dilution effect between samples and is considered the most useful approach for biomarker research in urine samples [73]. PQN, using the median of samples as a reference, was performed using the MetaboanalystR package version 4.0 [74]. In the same step, autoscaling was also applied.

Several approaches were followed to study the impact of the variable of study. Firstly, a univariate analysis (UVA) was performed to assess the effect of preterm birth at the metabolite level. The Mann–Whitney U test was used to compare the levels of each metabolite between the two groups. False Discovery Rate (FDR) correction was applied to adjust the *p*-value obtained and 0.05 was considered as the significance threshold. The fold change (FC, expressed as log_2_FC) was also calculated to determine the magnitude and direction of the change, considering the Term group as the reference. Both metrics (adjusted *p*-values and log_2_FC) were utilized to construct a volcano plot.

Next, the global impact of the variable of study was examined through multivariate analysis (MVA) using the ropls package version 1.32.0 [23]. Partial least squares discriminant analysis (PLS-DA) was used to determine the effect of preterm birth. Models were validated by permutation tests (200 permutations, threshold for pQ2 and pRY2 ≤ 0.05) and assessed using R2Y and Q2Y values. The most discriminant variables were identified based on the variable importance in projection (VIP) scores of the PLS algorithm. The cut-off threshold for significant compounds was determined using a normality plot, selecting the VIP value from which metabolites started to deviate from a normal distribution, as previously applied [75].

Finally, a hierarchical multiblock model was constructed to facilitate the biological interpretation of the results [76]. Briefly, each identified metabolite was assigned up to three biological functions (blocks) according to an in-house database built using open-access information. Blocks containing less than three metabolites were discarded. Next, an OPLS-DA model was created for each block. The scores for each individual on the principal component 1 (t1) for all models were obtained and weighted to account for the number of metabolites per block. A new PLS-DA model was also built to ensure that this process did not distort sample distribution compared to unblocked dataset.

A random forest test [77] was applied to determine the impact of the variable of interest on these generated blocks. Variable selection for the model was performed by plotting the mean decrease in Gini index on a normality graph. Blocks that deviated most from normal distribution were considered the functions most dysregulated between groups.

In addition, a pairwise partial correlation network analysis was performed to assess the impact of each function on the whole system. The R package GeneNet version 1.2.17 [78] was used to construct a single system for each group. Cytoscape software version 3.10.1 [25] was then applied to plot the minimal network for each group. For this, edges were filtered according to their *p*-values to obtain a system with the least number of interactions while including the whole set of nodes. Subsequently, the Term interactions were subtracted from the Prem network to obtain the interactions specific to the prematurity group. Blocks with the highest betweenness centrality in the constructed networks were considered to have the most impact on the biological system, as this metric indicates nodes with significant influence on the regulation of the entire network. The threshold was again chosen from a normality plot.

Additionally, PLS-DA models were constructed to assess the potential bias effects of several confounding variables on the whole metabolite set (e.g., infant sex, feeding pattern, type of delivery, single or multiple pregnancy and maternal gestational disease). In order to do so, Prem and Term group were subdivided in groups according to the corresponding factor and the resulting subgroups were compared in the MVA.

Analyses were performed in both techniques (LC-MS and NMR) separately and interpreted together.

## 5. Conclusions

In conclusion, the results herein presented contribute to a better understanding of the metabolic adaptations associated with preterm birth. The urine composition of extremely and very preterm infants greatly differs from that of full-term babies, reflecting the substantial metabolic impact of premature birth. Comprehensive metabolome analyses and profile characterization have established a robust connection between the preterm phenotype and several key biological functions. Notably, preterm birth was associated with significant shifts in nitrogen metabolism, growth, neurochemical metabolism, microbiota alterations, cell defense, and general metabolism alterations. These associations are supported by strong biological plausibility, providing valuable insights into the complex physiological landscape of premature infants. As a whole, the present work highlights the potential of urine, an easily obtainable biofluid, for studying systemic adjustments, offering a comprehensive metabolite profile that may reflect internal changes.

## Data Availability

The raw data supporting the conclusions of this article will be made available by the authors on request. During the preparation of this work, the authors used ChatGPT-4.0 (OpenAI) to assist with code development during data analysis and to improve the readability and language of the manuscript in the writing process. After using this tool/service, the author(s) reviewed and edited the content as needed and take full responsibility for the content of the published article.

## Supplementary Materials

The following supporting information can be downloaded at https://www.mdpi.com/article/10.3390/ijms26188953/s1.

## Abbreviations

BFP	Body Fat Percentage
CEI-IB	Comité de Ética de Investigación de las Islas Baleares
DOHaD	Developmental Origins of Health and Disease
FC	Fold Change
FDR	False Discovery Rate
HMDB	Human Metabolome Database
LC-MS	Liquid Chromatography coupled to Mass Spectrometry
MMP	Muscle Mass Percentage
MS/MS	Tandem Mass Spectrometry
MVA	Multivariate Analysis
NMR	Nuclear Magnetic Resonance
OPLS-DA	Orthogonal Partial Least Squares Discriminant Analysis
PLS-DA	Partial Least Squares Discriminant Analysis
PQN	Probabilistic Quotient Normalization
QC	Quality Control
TCA	Tricarboxylic acid cycle
UVA	Univariate Analysis
VIP	Variable Importance in Projection
