# Supplementary material for: Neonatal Urine Metabolic Signature Reflects Multisystemic Adaptations Linked to Preterm Birth
Source: Int J Mol Sci. 2025 Sep 14;26(18):8953. doi: 10.3390/ijms26188953 (PMC12469547; doi:10.3390/ijms26188953)
Supplement: Supplementary file 1 [file ijms-26-08953-s001.zip › Figure S3.pdf]

Figure S3

SEX EFFECT

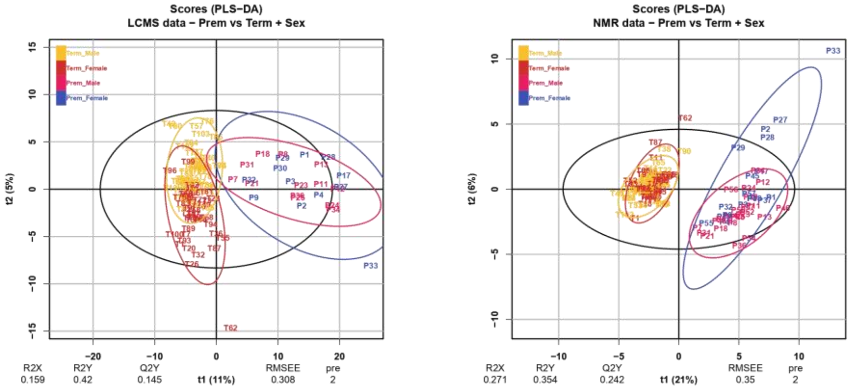

pR2Y = 0.005 pQ2 = 0.005

pR2Y = 0.005 pQ2 = 0.005

FEEDING PATTERNS EFFECT

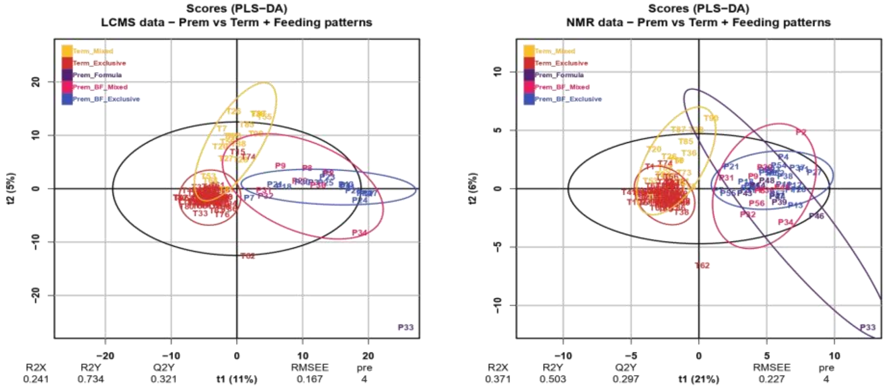

pR2Y = 0.005 pQ2 = 0.005

pR2Y = 0.005 pQ2 = 0.005

TYPE OF DELIVERY EFFECT

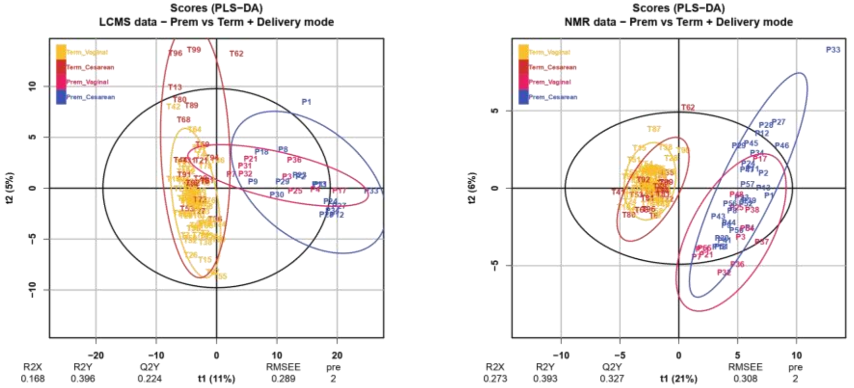

pR2Y = 0.005 pQ2 = 0.005

pR2Y = 0.005 pQ2 = 0.005

TYPE OF PREGNANCY EFFECT

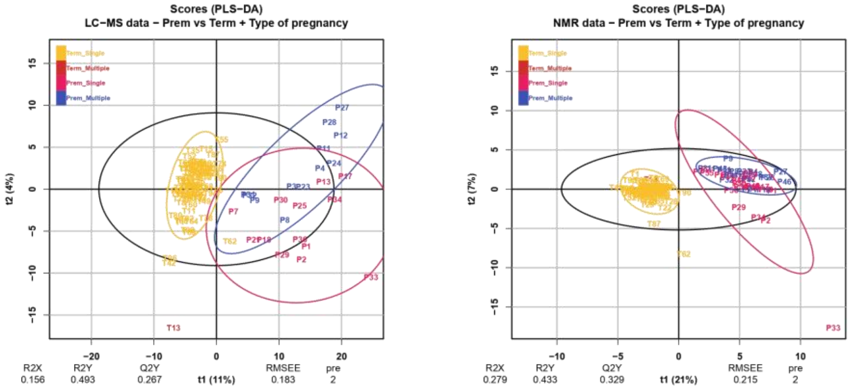

pR2Y = 0.005 pQ2 = 0.005

pR2Y = 0.005 pQ2 = 0.005

GESTATIONAL DISEASE EFFECT

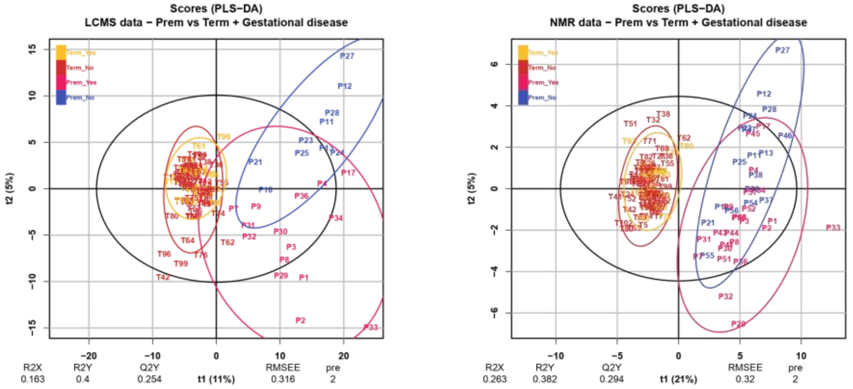

pR2Y = 0.005 pQ2 = 0.005

pR2Y = 0.005 pQ2 = 0.005
