# Supplementary material for: Neonatal Urine Metabolic Signature Reflects Multisystemic Adaptations Linked to Preterm Birth
Source: Int J Mol Sci. 2025 Sep 14;26(18):8953. doi: 10.3390/ijms26188953 (PMC12469547; doi:10.3390/ijms26188953)

Figure S5

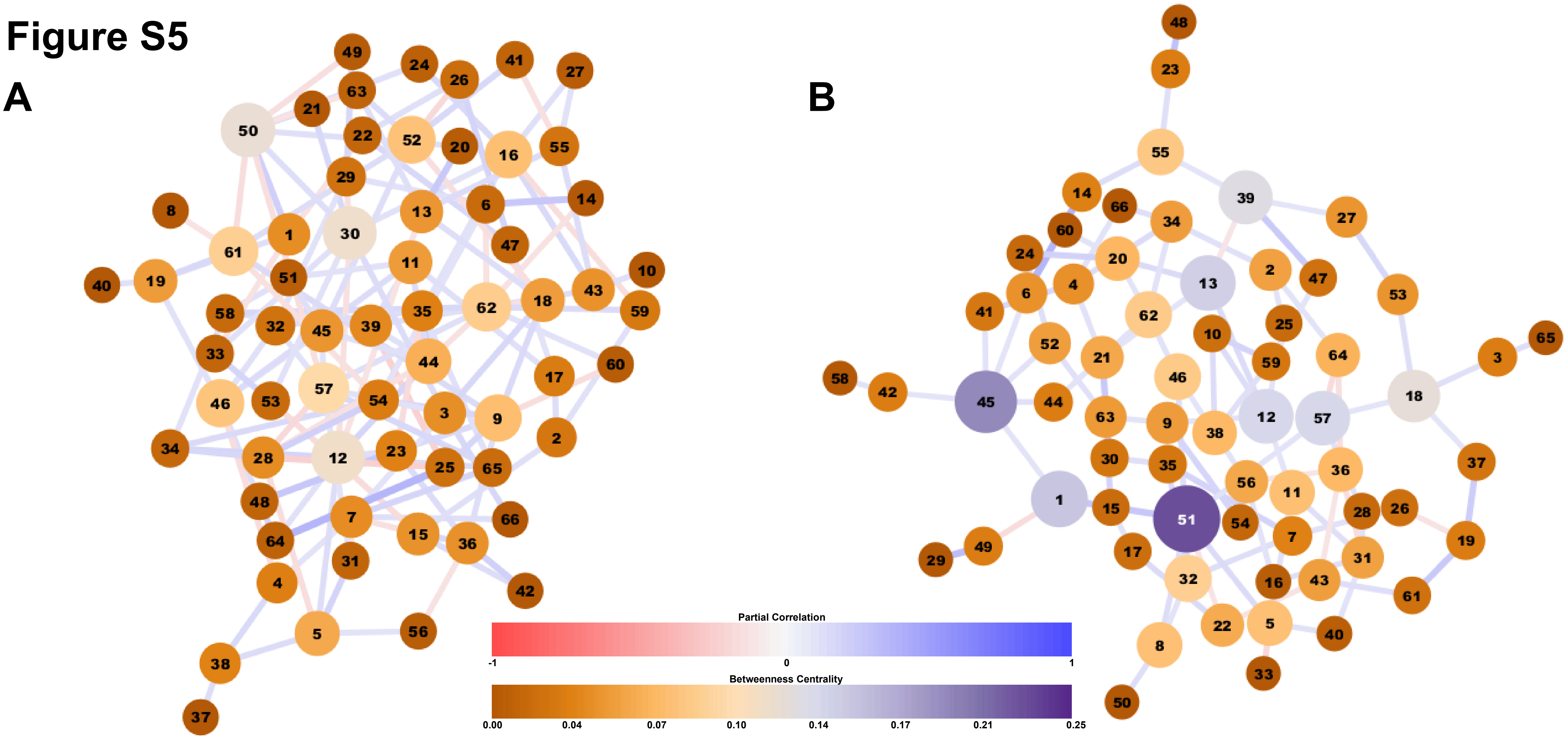

1, Dipeptides; **2**, Aminoacids metabolism; **3**, Antiinflammatory; **4**, Antioxidant; **5**, Arginine metabolism; **6**, Bile acid metabolism; **7**, Branched-chain aminoacids metabolism; **8**, CHO metabolism; **9**, Caffeine metabolism; **10**, Catecholamines metabolism; **11**, Cell growth and proliferation; **12,** Cell signaling; **13**, Cell transduction and communication; **14**, Cholesterol metabolism; **15**, Collagen metabolism; **16**, Cystein and homocystein metabolism; **17**, Detoxication; **18**, Drug; **19**, Energy metabolism; **20**, Epigenetic modification; **21**, Fatty acid B-oxidation; **22**, Fatty acid metabolism; **23**, Ganglioside metabolism; **24**, Gene expression; **25**, General metabolism; **26**, Gut function; **27**, Immune function; **28**, Inflammation; **29**, Kidney function; **30**, Lipid metabolism; **31**, Metabolic activation; **32**, Metabolic regulation; **33**, Metabolic intermediates; **34**, Microbiota metabolism; **35**, Mitochondrial function; **36**, Multiple disorders; **37**, Muscle metabolism; **38**, NO metabolism; **39**, Neurological dysfunction; **40**, Neuronal metabolism and neurotransmitters; **41**, Neuroprotection; **42**, Neurotoxicity; **43**, Neurotransmitter; **44**, Nucleic acid metabolism; **45**, Nucleotide metabolism; **46**, Oxidative stress; **47**, Phenylalanine metabolism; **48**, Phospholipid metabolism; **49**, Post-translational regulation; **50**, Prebiotic; **51**, Protein metabolism; **52**, Purine metabolism; **53**, Pyrimidine metabolism; **54**, Redox status; **55**, Steroid metabolism; **56**, Stress response; **57**, TCA; **58**, Tryptophan metabolism; **59**, Tyrosine metabolism; **60**, Unknown; **61**, Urea cycle; **62**, Uremic toxins; **63**, Vascular health; **64**, Vitamin metabolism; **65**, W-oxidation; **66**, Xenobiotics

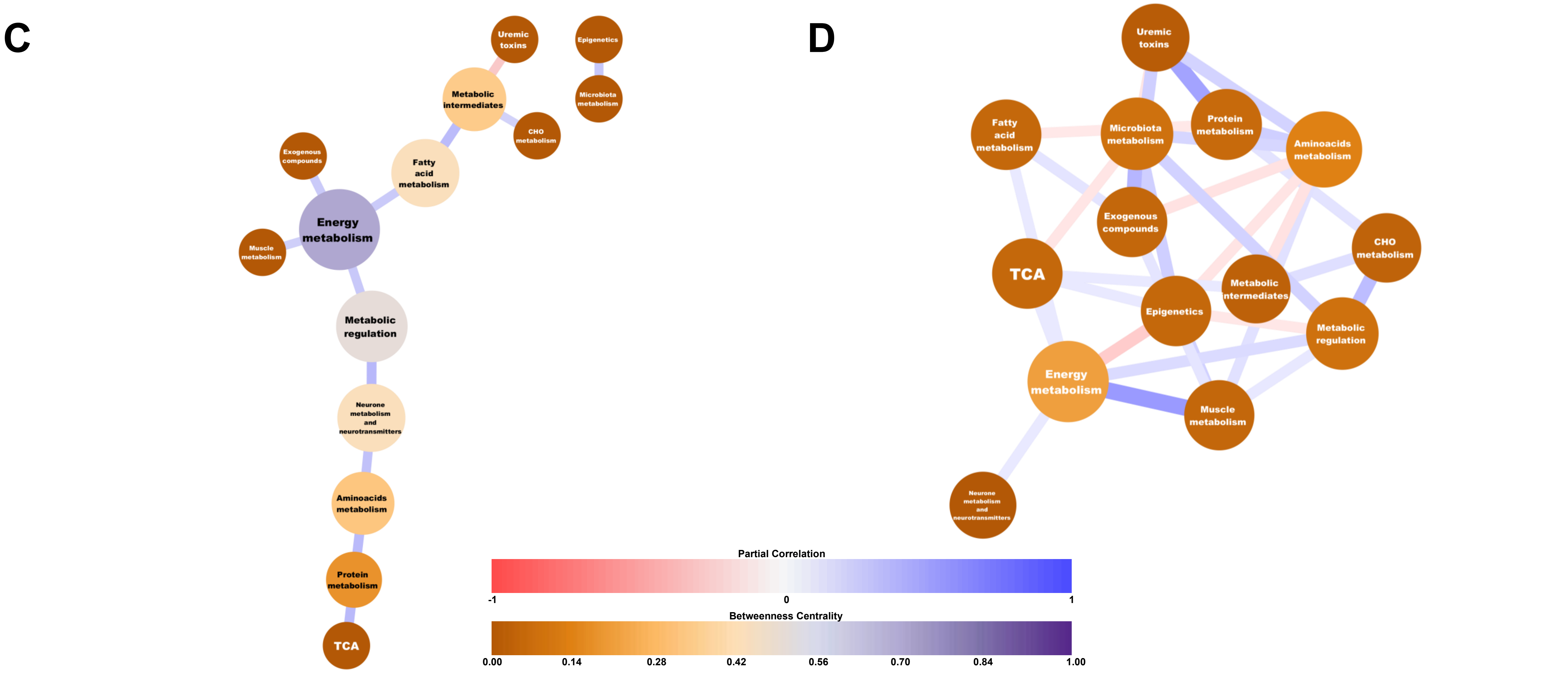

Supplement: Supplementary file 1 [file ijms-26-08953-s001.zip › Figure S5.pdf]
