# Supplementary material for: Neonatal Urine Metabolic Signature Reflects Multisystemic Adaptations Linked to Preterm Birth
Source: Int J Mol Sci. 2025 Sep 14;26(18):8953. doi: 10.3390/ijms26188953 (PMC12469547; doi:10.3390/ijms26188953)
Supplement: Supplementary file 1 [file ijms-26-08953-s001.zip › Supplementary tables.pdf]

**Supplementary table S1**. Relevant characteristics of the mothers of the study included in the metabolomics analyses.

| Group | Prem | Term | p-value | Effect size |
|---|---|---|---|---|
| Observations | 45 | 96 | | |
| Ethnicity | | | | |
| Caucasian | 64% (29) | 76% (73) | **0.049** | **0.264** |
| Asiatic | 0% (0) | 4.2% (4) | | |
| Latin American | 11% (5) | 16% (15) | | |
| African | 11% (5) | 4.2% (4) | | |
| Romany | 4.4% (2) | 0% (0) | | |
| Missing | 8.9% (4) | 0% (0) | | |
| Tobacco | | | | |
| Never | 87% (39) | 96% (92) | 0.350 | 0.177 |
| <2-3 cigarettes/day | 2.2% (1) | 1% (1) | | |
| <5 cigarettes/day | 4.4% (2) | 1% (1) | | |
| 5-9 cigarettes/day | 2.2% (1) | 2.1% (2) | | |
| 10-20 cigarettes/day | 2.2% (1) | 0% (0) | | |
| Missing | 2.2% (1) | 0% (0) | | |
| Alcohol | | | | |
| Occasional | 0% (0) | 6.2% (6) | 0.210 | 0.143 |
| Never | 98% (44) | 94% (90) | | |
| Missing | 2.2% (1) | 0% (0) | | |
| Drugs | | | | |
| Yes | 2.2% (1) | 0% (0) | 0.690 | 0.125 |
| No | 96% (43) | 100% (96) | | |
| Missing | 2.2% (1) | 0% (0) | | |
| Previous lactation | | | | |
| Yes | 38% (17) | 47% (45) | 0.690 | 0.050 |
| No | 53% (24) | 53% (51) | | |
| Missing | 8.9% (4) | 0% (0) | | |
| Number of previous pregnancies | | | | |
| 0 | 38% (17) | 43% (41) | 0.300 | 0.186 |
| 1 | 36% (16) | 40% (38) | | |
| 2 | 8.9% (4) | 11% (11) | | |
| 3 | 16% (7) | 5.2% (5) | | |
| 6 | 0% (0) | 1% (1) | | |
| Missing | 2.2% (1) | 0% (0) | | |
| Pregnancy complications | | | | |
| None | 32% (12) | 77% (74) | **<0.001** | **0.660** |
| Endocrine disorders | 21% (8) | 11% (11) | | |
| Hypertension disorders | 24% (9) | 3% (3) | | |
| Obstetric disorders | 16% (6) | 1% (1) | | |
| Infections | 8% (3) | 7% (7) | | |
| Type of pregnancy | | | | |
| Multiple | 49% (22) | 1% (1) | **<0.001** | **0.613** |
| Single | 49% (22) | 99% (95) | | |
| Missing | 2.2% (1) | 0% (0) | | |
| Type of delivery | | | | |
| Cesarean | 67% (30) | 24% (23) | **<0.001** | **0.423** |
| Natural | 31% (14) | 76% (73) | | |
| Missing | 2.2% (1) | 0% (0) | | |
| Breastfeeding | | | | |
| Yes | 80% (36) | 100% (96) | **<0.001** | **0.344** |
| No | 16% (7) | 0% (0) | | |
| Missing | 4.4% (2) | 0% (0) | | |
| Type of breastfeeding | | | | |
| Mixed | 22% (10) | 18% (17) | 0.260 | 0.119 |
| Exclusive | 56% (25) | 82% (79) | | |
| Missing | 22% (10) | 0% (0) | | |

Statistical analysis applied in Prematurity vs Term contrasts: Fisher exact test. Effect size assessed with Cramer's V statistic using the Term group as the reference.
Values in bold correspond to statistically significant contrasts.

**Supplementary table S2.** Anthropometric characteristics of the mothers of the study included in the metabolomics analyses.

| Group | Prem | Term | p-value | Effect size |
|---|---|---|---|---|
| Observations | 45 | 96 | | |
| Age (years) | | | | |
| Median | 36 | 36 | 0.780‡ | -0.091 |
| Mean | 34.9 | 35.4 | | |
| SD | 5.94 | 4.54 | | |
| Gestational age (weeks) | | | | |
| Median | 30.2 | 39.9 | **<0.001‡** | **-7.036** |
| Mean | 30.1 | 39.6 | | |
| SD | 1.59 | 1.23 | | |
| preBMI ($kg/m^2$) | | | | |
| Median | 22.9 | 22.9 | 0.539‡ | -0.1159 |
| Mean | 23.5 | 24.1 | | |
| SD | 4.25 | 4.56 | | |
| GWG (kg) | | | | |
| Median | 10.3 | 12 | 0.104‡ | -0.166 |
| Mean | 11.0 | 11.8 | | |
| SD | 5.22 | 4.36 | | |
| BMI ($kg/m^2$) | | | | |
| Median | 24.3 | 24.2 | 0.411‡ | -0.124 |
| Mean | 24.8 | 25.3 | | |
| SD | 4.59 | 4.44 | | |
| BFP (%) | | | | |
| Median | 32.1 | 37.5 | **0.007#** | **-0.558** |
| Mean | 33.4 | 37.3 | | |
| SD | 7.68 | 6.78 | | |
| VF (%) | | | | |
| Median | 5 | 5.5 | 0.408‡ | -0.218 |
| Mean | 5.26 | 5.64 | | |
| SD | 1.89 | 1.67 | | |
| MMP (%) | | | | |
| Median | 28.6 | 25.7 | **<0.001#** | **0.792** |
| Mean | 28.3 | 26.1 | | |
| SD | 3.13 | 2.62 | | |

Statistical analysis applied in Prematurity vs Term contrasts: T-test for variables with a normal distribution (#), Mann-Whitney U for non-parametric variables (‡) (checked with Lilliefors test). Effect size assessed with Cohen's D statistic using the Term group as the reference.
SD, standard error of the mean; preBMI, body mass index before pregnancy; GWG, gestational weight gain; BMI, body mass index; BFP, body fat percentage; MMP, muscle mass percentage; VF, visceral fat. Values in bold correspond to statistically significant contrasts.

**Supplementary table S3.** Somatometric parameters of the infants of the study included in the metabolomics analyses.

| Group | Prem | Term | p-value | Effect size |
|---|---|---|---|---|
| | | | **All** | |
| Observations | 45 | 96 | | |
| Sex | | | | |
| Female | 18 | 43 | 0.716 | 0.036 |
| Male | 26 | 53 | | |
| Missing | 1 | 0 | | |
| Subclass of preterm birth | | | | |
| Very preterm | 39 | - | | |
| Extremely preterm | 5 | - | | |
| Missing | 1 | - | | |
| Birth weight (kg) | | | | |
| Median | 1.24 | 3.36 | **<0.001‡** | **-5.33** |
| Mean | 1.25 | 3.31 | | |
| SD | 0.298 | 0.421 | | |
| p Birth weight | | | | |
| Median | 29 | 58 | **0.001‡** | **-0.607** |
| Mean | 35.27 | 53.08 | | |
| SD | 30.3 | 28.86 | | |
| Birth length (cm) | | | | |
| Median | 38.5 | 50 | **<0.001‡** | **-5.34** |
| Mean | 38.1 | 50.0 | | |
| SD | 3.03 | 1.74 | | |
| p Birth length | | | | |
| Median | 18.5 | 57 | **<0.001‡** | **-0.648** |
| Mean | 33.3 | 53.0 | | |
| SD | 34.0 | 28.6 | | |
| Birth head circumference (cm) | | | | |
| Median | 27.2 | 35 | **<0.001‡** | **-5.05** |
| Mean | 27.23 | 34.72 | | |
| SD | 2.082 | 1.113 | | |
| p Birth head circumference | | | | |
| Median | 45 | 51 | 0.231‡ | -0.245 |
| Mean | 46.4 | 52.7 | | |
| SD | 31.21 | 23.02 | | |
| Weight at one month (kg) | | | | |
| Median | 1.79 | 4.26 | **<0.001‡** | **-4.56** |
| Mean | 1.83 | 4.31 | | |
| SD | 0.451 | 0.581 | | |
| p Weight at one month | | | | |
| Median | 8.35 | 54.5 | **<0.001‡** | **-1.36** |
| Mean | 18.8 | 54.4 | | |
| SD | 23.44 | 27.32 | | |
| Length at one month (cm) | | | | |
| Median | 42 | 54.5 | **<0.001‡** | **-4.35** |
| Mean | 41.9 | 54.3 | | |
| SD | 3.73 | 2.34 | | |
| p Length at one month | | | | |
| Median | 12.5 | 62 | **<0.001‡** | **-1.60** |
| Mean | 19.7 | 61.5 | | |
| SD | 25.17 | 26.66 | | |
| Head circumference at one month (cm) | | | | |
| Median | 30 | 37.5 | **<0.001‡** | **-3.91** |
| Mean | 30.3 | 37.4 | | |
| SD | 2.84 | 1.11 | | |
| p Head circumference at one month | | | | |
| Median | 17.5 | 79 | **<0.001‡** | **-1.68** |
| Mean | 28.73 | 71.69 | | |
| SD | 27.21 | 24.78 | | |
| Body weight gain (kg) | | | | |
| Median | 0.57 | 0.98 | **<0.001‡** | **-1.09** |
| Mean | 0.580 | 0.994 | | |
| SD | 0.225 | 0.431 | | |
| p Weight increment | | | | |
| Median | -9 | 1.5 | **<0.001#** | **-0.730** |
| Mean | -16.0 | 1.3 | | |
| SD | 22.93 | 24.04 | | |
| Length gain (cm) | | | | |
| Median | 4 | 4 | 0.189‡ | -0.271 |
| Mean | 3.77 | 4.27 | | |
| SD | 2.22 | 1.62 | | |
| p Length increment | | | | |

| Group | Prem | Term | p-value | Effect size |
|---|---|---|---|---|
| Median | -5.5 | 8 | **<0.001#** | **-0.87** |
| Mean | -13.5 | 8.02 | | |
| SD | 27.26 | 23.52 | | |
| Head circumference increment (cm) | | | | |
| Median | 2.5 | 3 | 0.755‡ | 0.214 |
| Mean | 3.04 | 2.70 | | |
| SD | 2.36 | 1.057 | | |
| p Head circumference increment | | | | |
| Median | -17 | 22 | **<0.001#** | **-1.282** |
| Mean | -17.9 | 19.06 | | |
| SD | 29.09 | 28.72 | | |

Statistical analysis applied in Prematurity vs Term contrasts for categorical variables (sex): Fisher exact test. Effect size assessed with Cramer's V statistic using the Term group as the reference. Statistical analysis applied in Prematurity vs Term contrasts for numerical variables: T-test for variables with a normal distribution (#), Mann-Whitney U for non-parametric variables (‡) (checked with Lilliefors test). Effect size assessed with Cohen's D statistic using the Term group as the reference. Values in bold correspond to statistically significant contrasts.
SD, standard error of the mean; p, percentile adjusted for gestational age

**Supplementary table S4.** List of metabolites identified in urine samples, according to the metabolomic approach followed. For each compound, both names and HMDB ID are presented (if available). In RMN technique, UX.XX indicates an unknown ROI (U) and the chemical shift at which it was detected (X.XX, in ppm).

| Metabolites identified solely with RMN technique (n=36) | | | |
|---|---|---|---|
| **Compound name** | **HMDB ID** | **Compound name** | **HMDB ID** |
| 1-Methylnicotinamide | HMDB0000699 | p-Hydroxyphenylacetic acid | HMDB0000020 |
| 3-Hydroxyisobutyric acid | HMDB0000023 | Propylene Glycol | HMDB0001881 |
| 3-Hydroxyisovaleric acid | HMDB0000754 | Pseudouridine | HMDB0000767 |
| 4-DEA | HMDB0000498 | Sarcosine | HMDB0000271 |
| 4-DTEA | HMDB0002453 | Trimethylamine | HMDB0000906 |
| Acetic acid | HMDB0000042 | U0.765 | |
| Acetone | HMDB0001659 | U1.05 | |
| Alpha-Hydroxyisobutyric acid | HMDB0000729 | U1.80 | |
| Benzoic acid | HMDB0001870 | U1.99 | |
| D-Galactose | HMDB0000143 | U2.30 | |
| Dimethylamine | HMDB0000087 | U2.76 | |
| Dimethylglycine | HMDB0000092 | U2.79 | |
| Ethanol | HMDB0000108 | U2.86 | |
| Formic acid | HMDB0000142 | U3.112 | |
| Lactic-acid/L-Threonine | HMDB0000190/ HMDB0000167 | U4.11 | |
| L-Alanine | HMDB0000161 | U4.65 | |
| Methanol | HMDB0001875 | U5.54 | |
| N-Methylnicotinamide | HMDB0003152 | Urea | HMDB0000294 |
| **Metabolites identified with both techniques (n=38)** | | | |
| **Compound name** | **HMDB ID** | **Compound name** | **HMDB ID** |
| 1-Methylhistidine | HMDB0000001 | L-Glutamine | HMDB0000641 |
| 3-Indoxylsulfate | HMDB0000682 | L-Histidine | HMDB0000177 |
| 3-Methyladipic acid | HMDB0000555 | L-Isoleucine | HMDB0000172 |
| 4-Hydroxyproline | HMDB0000725 | L-Leucine | HMDB0000687 |
| Alpha-Lactose | HMDB0000186 | L-Lysine | HMDB0000182 |
| Betaine | HMDB0000043 | L-Tryptophan | HMDB0000929 |
| Choline | HMDB0000097 | L-Tyrosine | HMDB0000158 |
| cis-Aconitic acid | HMDB0000072 | L-Valine | HMDB0000883 |
| Citric acid | HMDB0000094 | Methylguanidine | HMDB0001522 |
| Creatine | HMDB0000064 | Myoinositol | HMDB0000211 |
| Creatinine | HMDB0000562 | N-Acetyl-L-Aspartic acid | HMDB0000812 |
| D-Glucose | HMDB0003345 | Oxoglutaric acid | HMDB0000208 |
| D-Xylose | HMDB0000098 | Phenylacetylglycine | HMDB0000821 |
| Fumaric Acid | HMDB0000134 | Pyruvic acid | HMDB0000243 |
| Gluconic acid | HMDB0000625 | Succinic acid | HMDB0000254 |
| Glycine | HMDB0000123 | Sucrose | HMDB0000258 |
| Hippuric acid | HMDB0000714 | Taurine | HMDB0000251 |
| L-Carnitine | HMDB0000062 | Trimethylamine-N-oxide | HMDB0000925 |
| L-Glutamic acid | HMDB0000148 | Xanthosine | HMDB0000299 |
| **Metabolites identified solely with LC-MS technique (n=483)** | | | |
| **Compound name** | **HMDB ID** | **Compound name** | **HMDB ID** |
| 2-Methylglutarate | HMDB0000422 | Galacturonate | |

| | | | |
|---|---|---|---|
| 3,4-Dihydroxyhydrocinnamic acid | HMDB0000423 | Gallic acid | HMDB0005807 |
| (-)-Cotinine | HMDB0001046 | Gamma-Aminobutyrate | HMDB0000112 |
| 2-Oxoadipate | HMDB0000225 | Gamma-Hydroxybutyric acid | HMDB0000710 |
| 3-(3,4-Dihydroxyphenyl)Lactate | HMDB0003503 | Gluconolactone | HMDB0000150 |
| 2-Ethylsuberic acid | HMDB0059708 | Glucosamine | HMDB0001514 |
| 21-Deoxycortisol | HMDB0004030 | Glucose 1-Phosphate | HMDB0001586 |
| 2'-O-Methyladenosine | HMDB0004326 | Glucose 6-Phosphate | HMDB0001401 |
| 2-Oxobutanoic acid | HMDB0000005 | Glucuronate | HMDB00127 |
| 2,6-Quinolinediol | | Glutarate | HMDB0000661 |
| 3-(2,4-Dihydroxyphenyl)Propionic acid | HMDB0126386 | Glu-Val | HMDB0028832 |
| 2-Hydroxydodecanoic acid | HMDB0245154 | Glycerate | HMDB0000139 |
| 2-Hydroxycaproic acid | HMDB0001624 | Glycerol 3-Phosphate | HMDB0000126 |
| 2-Methoxy-4-Vinylphenol | HMDB0013744 | Guanidinosuccinate | HMDB0003157 |
| 2-Hydroxy 3-Methylbutyric acid | HMDB0000407 | Guanine | HMDB0000132 |
| 2-Aminophenol | | Guanosine | HMDB0000133 |
| 3-(3,5-Dihydroxyphenyl)Propanoic acid | HMDB0125533 | Heptylmalonic acid | HMDB0059719 |
| Nordeoxycholic acid | HMDB0304947 | Hexanoic acid | HMDB0000535 |
| 1-Methyl-Pyrogallol-3-O-Sulfate | HMDB0341060 | Hexanoylglycine | HMDB0000701 |
| 1-Methyltryptophan | HMDB0243932 | Hexanoyl-L-Carnitine | HMDB0000756 |
| 2-Methoxyresorcinol | HMDB0133970 | Hinokitiol | |
| (E)-3-Methylglutaconic acid | HMDB0000522 | Histamine | HMDB0000870 |
| (4R)-5-(3,4-Dihydroxyphenyl)-Gamma-Valerolactone-4'-O-Sulfate | HMDB0029185 | Histidinol | HMDB0003431 |
| 1-Methyladenosine | HMDB0003331 | H-Leu-Ile-Oh | HMDB0028932 |
| 2-Methylhippuric acid | HMDB0011723 | H-Leu-Pro-Oh | HMDB0011175 |
| 3-(3-Hydroxyphenyl)Propionic acid | HMDB0000375 | H-Leu-Tyr-Oh | HMDB0028941 |
| (R)-(-)-Phenylephrine | HMDB0002182 | Homocysteine | HMDB0000676 |
| 2,3-Diaminopropionate | HMDB0002006 | Homogentisic acid | HMDB0000130 |
| 3-(3,4,5-Trimethoxyphenyl)Propionic acid | HMDB0030254 | Homovanillate | HMDB0000118 |
| 2-Hydroxy-4-Phenylbutyric acid | | Homovanillic acid Sulfate | HMDB0011719 |
| 2-Quinolinecarboxylate | HMDB0000842 | Homoveratric acid | HMDB0000434 |
| 3,4-Dihydroxybenzaldehyde | HMDB0059965 | Hydroxykynurenine | HMDB0000732 |
| 2,4-Dihydroxybenzaldehyde | HMDB0062484 | Hydroxyphenyllactate | HMDB0000755 |
| 2,4,6-Trihydroxybenzoic acid | HMDB0029649 | Hydroxytyrosol | HMDB0005784 |
| 2-Hydroxypyridine | HMDB0013751 | Hypotaurine | HMDB0000965 |
| 3-(2-Hydroxyphenyl)Propanoate | HMDB0033752 | Imidazolepropionic acid | HMDB0002271 |
| 2-Acetamido-2-Deoxy-Beta-D-Glucosylamine | | Indole | HMDB0000738 |
| 2-Isopropylmalic acid | HMDB0000402 | Indole-3-Acetate | HMDB0000197 |
| 2-Hydroxybenzyl Alcohol | HMDB0059709 | Indole-3-Acetic acid Glucuronide | HMDB0060001 |
| (S)-3-Hydroxybutyric acid | HMDB0000442 | Indole-3-Acryloylglycine | HMDB0006005 |
| 14-0 Lysope | HMDB0011500 | Indole-3-Carboxaldehyde | HMDB0029737 |
| 1,3-Diaminopropane | HMDB0000002 | Indole-3-Ethanol | HMDB0003447 |
| 10-Hydroxyoctadecanoic acid | HMDB0037396 | Indoleacetyl Glutamine | HMDB0013240 |
| 1-Methyluric acid | HMDB0003099 | Indoxyl Beta-D-Glucuronide | HMDB0010319 |
| 17A-Hydroxypregnenolone | HMDB0000363 | Inosine | HMDB0000195 |
| 2'-O-Methylinosine | HMDB0341419 | Isodehydracetic acid | |
| 2-Hydroxyisocaproic acid | HMDB0000746 | Isoferulic acid-3-O-Glucuronide | HMDB0041747 |
| 2,4-Dihydroxycinnamic acid | | Isonicotinic acid N-Oxide | |

| | | | |
|---|---|---|---|
| 2,6-Dihydroxybenzoic acid | HMDB0013676 | Isovaleroylglycine | HMDB0000678 |
| 1,7-Dimethyluric acid | HMDB0011103 | Isovaleryl-L-Carnitine | HMDB0000688 |
| 2-Oxo-4-Phenyl-Butyric acid | | Kynurenic acid | HMDB0000715 |
| 16:0 Lysopa | HMDB0007853 | Kynurenine | HMDB0000684 |
| 17A-Hydroxyprogesterone | HMDB0000374 | L-Arginine | HMDB0000517 |
| 2-Aminoisobutyrate | HMDB0001906 | L-Asparagine | HMDB0000168 |
| 3,4-Dihydroxyphenylvaleric acid | HMDB0029233 | L-Beta-Homoisoleucine Hydrochloride | |
| 3-(3-Hydroxyphenyl)-3-Hydroxypropionic acid | HMDB0002643 | L-Beta-Homomethionine-Hcl | |
| 1,5-Anhydro-D-Sorbitol | HMDB0002712 | L-Beta-Homophenylalanine Hydrochloride | |
| 2-Hydroxy-4-(Methylthio)Butanoate | HMDB0037115 | L-Gulonolactone | HMDB0003466 |
| 2,2-Dimethylglutaric acid | HMDB0061676 | Linoleic acid | HMDB0000673 |
| 2',4'-Dihydroxyacetophenone | HMDB0029659 | Ll-2,6-Diaminoheptanedioate | HMDB0001370 |
| 3,3-Dimethylglutaric acid | HMDB0002441 | L-Methionine | HMDB0000696 |
| 3,4-Dihydroxymandelate | HMDB0001866 | L-Methionine Sulfone | HMDB0062174 |
| 3,4-Dihydroxybenzenesulfonic acid | | L-Ornithine | HMDB0000214 |
| 3,4-Dihydroxybenzoate | HMDB0001856 | L-Phenylalanine | HMDB0000159 |
| 3,4-Dihydroxy-L-Phenylalanine | HMDB0000181 | L-Serine | HMDB0000187 |
| 3,4-Dihydroxyphenylacetate | HMDB0001336 | L-Threo-3-Phenylserine | HMDB0002184 |
| 3,4-Dihydroxyphenylglycol | HMDB0000318 | L-Threonine | HMDB0000167 |
| 3,5-Dihydroxybenzoic acid | HMDB0013677 | Lumichrome | HMDB0254199 |
| Tetrahydrocortisol | HMDB0000949 | Lyxose | HMDB0003402 |
| 3-Amino-4-Hydroxybenzoate | HMDB0304941 | Malate | HMDB0000156 |
| 3-Ethoxy-4-Hydroxybenzaldehyde | HMDB0029665 | Maleamate | HMDB0254310 |
| 3-Hydroxy-7-Oxo-5-Cholanic acid | HMDB0000467 | Maleate | HMDB0000176 |
| 2-Indolecarboxylic acid | HMDB0002285 | Malonate | HMDB0000691 |
| 12-Ketochenodeoxycholic acid | HMDB0000400 | Maltose | HMDB0000163 |
| 3-Hydroxy-7,12-Diketocholanoic acid | | Maltotriose | HMDB0001262 |
| 3-Hydroxyanthranilate | HMDB0001476 | Mannitol | HMDB0000765 |
| 3-Hydroxybenzaldehyde | | M-Cresol Sulfate | |
| 3-Hydroxybenzoate | HMDB0002466 | Melibiose | HMDB0000048 |
| 3-Hydroxybenzyl Alcohol | HMDB0059712 | Meso-Tartrate | HMDB0062642 |
| 3-Hydroxybutanoate | HMDB0000011 | Mesoxalate | HMDB0031522 |
| 3-Hydroxymandelic acid | HMDB0000750 | Methanesulfonic acid | HMDB0240280 |
| 3-Hydroxymethylglutarate | HMDB0000355 | Methyl Galactoside | |
| 3-Hydroxyphenylacetate | HMDB0000440 | Methyl-Glucuronide | |
| 3-Hydroxyphenylvaleric acid | HMDB0041666 | Methylglutarylcarnitine | HMDB0000552 |
| 3-Indole Carboxylic acid Glucuronide | HMDB0013189 | Methylmalonic acid | HMDB0000202 |
| 3-Indolepropionic acid | HMDB0002302 | Methyl-Succinic acid | HMDB0253609 |
| 3-Isopropylmalic acid | HMDB0012156 | Mevalolactone | HMDB0006024 |
| 3-Methoxycatechol | | M-Hydroxyhippuric acid | HMDB0006116 |
| 3-Methoxytyramine | HMDB0000022 | Mono-Methyl Adipate | HMDB0059722 |
| 3-Methoxytyrosine | HMDB0001434 | N(6)-Carboxymethyllysine | HMDB0240347 |
| 3-Methyl-2-Oxindole | HMDB0304943 | N,N,N-Trimethyllysine | HMDB0001325 |
| 3-Methyl-2-Oxovalerate | HMDB0000491 | N,N-Dimethyl-1,4-Phenylenediamine | HMDB0247696 |
| 3-Methyladenine | HMDB0011600 | N,N-Dimethylguanosine | HMDB0004824 |
| 3-Methylcrotonyl-L-Carnitine | | N2-Acetylaminoadipic acid | HMDB0062715 |
| 3-Methylglutaric acid | HMDB0000752 | N2-Methylguanosine | HMDB0005862 |

| | | | |
|---|---|---|---|
| 3-Methylhippuric acid | HMDB0013245 | N6-(Delta2-Isopentenyl)-Adenine | |
| 3-Methylhistamine | HMDB0001861 | N-Acetyl-Alanine | HMDB0000766 |
| 3-Methylindole | HMDB0000466 | N-Acetyl-Asparagine | HMDB0006028 |
| 3-Nitro-L-Tyrosine | HMDB0001904 | N-Acetyl-Cysteine | HMDB0001890 |
| 3-Phenyllactic acid | HMDB0000748 | N-Acetyl-Dl-Valine | HMDB0011757 |
| 3-Sulfinoalanine | HMDB0000996 | N-Acetyl-Galactosamine | HMDB0000212 |
| 4-(4-Hydroxyphenyl)Butan-2-One | HMDB0033723 | N-Acetyl-Glucosamine | HMDB0000803 |
| 4-Acetamidobenzoic acid | HMDB0246328 | N-Acetyl-Glutamate | HMDB0001138 |
| 4-Acetamidobutanoate | HMDB0003681 | N-Acetyl-Glycine | HMDB0000532 |
| 4-Aminobenzoate | HMDB0304171 | N-Acetyl-Leucine | HMDB0011756 |
| 4-Aminophenyl Hydrogen Sulfate | | N-Acetyl-L-Tyrosine | HMDB0000866 |
| 4-Ethylphenyl Sulfate Ammonium | | N-Acetyl-Mannosamine | HMDB0001129 |
| 4-Guanidinobutanoate | HMDB0003464 | N-Acetyl-Methionine | HMDB0011745 |
| 4-Hydroxy-3-Methylbenzoic acid | HMDB0004815 | N-Acetyl-Neuraminate | HMDB0000230 |
| 4-Hydroxybenzaldehyde | HMDB0011718 | N-Acetyl-Phenylalanine | HMDB0000512 |
| 4-Hydroxybenzoic acid | HMDB0000500 | N-Acetyl-Putrescine | HMDB0002064 |
| 4-Hydroxyhippuric acid | HMDB0013678 | N-Acetyl-Serine | HMDB0002931 |
| 4-Hydroxy-L-Phenylglycine | HMDB0244973 | N-Acetyl-Serotonin | HMDB0001238 |
| 4-Imidazoleacetate | HMDB0002024 | N-Acetyl-Tryptophan | HMDB0013713 |
| 4-Methyl-2-Oxovaleric acid | HMDB0000695 | N-Alpha-Acetyllysine | HMDB0000446 |
| 4-Methylcatechol | HMDB0000873 | N-Carbamyl-L-Glutamic acid | HMDB0015673 |
| 4-Methylcatechol Monosulfate | HMDB0240459 | N-Formyl-L-Methionine | HMDB0001015 |
| 4-Methylhippuric acid | HMDB0013292 | Nicotinamide Mononucleotide | HMDB0000229 |
| 4-Pyridoxate | HMDB0000017 | Nicotinic acid | HMDB0001488 |
| 4-Quinolinecarboxylic acid | HMDB0257047 | N-Methyl-2-Pyridone-5-Carboxamide | |
| 5-Aminolevulinate | HMDB0001149 | N-Methyl-Aspartate | HMDB0002393 |
| 5-Aminopentanoate | HMDB0003355 | N-Methyl-Dl-Alanine | HMDB0255173 |
| 5B-Cholanic acid-3A,6A,7A-Triol | HMDB0000415 | N-Methyl-Glutamate | HMDB0062660 |
| 5'-Deoxyadenosine | HMDB0001983 | Nonanoylcarnitine | HMDB0013288 |
| 5-Hydroxyindole | HMDB0059805 | Normetanephrine | HMDB0000819 |
| 5-Hydroxyindole-3-Acetic acid | HMDB0000763 | Norvaline | HMDB0013716 |
| 5-Hydroxylysine | HMDB0000450 | N-Phenylacetyl-Methionine | |
| 5-Hydroxymethyl-2-Furancarboxylic acid | HMDB0002432 | O-Acetyl-L-Carnitine | HMDB0240773 |
| 5-Hydroxytryptophan | HMDB0000472 | O-Acetyl-L-Serine | HMDB0003011 |
| 5-Methoxyindoleacetic acid | HMDB0004096 | O-Cresol Sulfate | HMDB0240655 |
| 5-Methylcytosine | HMDB0002894 | Octanoic acid Glucuronide | |
| 5-Methylthioribose | HMDB0001087 | Octanoyl-L-Carnitine | HMDB0000791 |
| 7-(1-Carboxyethyl)Lysine | | Octenoyl-L-Carnitine | HMDB0013324 |
| 7,8-Dihydro-L-Biopterin | HMDB0000038 | O-Hydroxyhippuric acid | HMDB0000840 |
| 7-Ketodeoxycholic acid | HMDB0000391 | Omega-Hydroxydodecanoate | HMDB0002059 |
| 7-Methylguanine | HMDB0000897 | O-Methylsinapic acid | HMDB0002511 |
| 7-Methylxanthine | HMDB0001991 | Ophthalmate | HMDB0005765 |
| Aamu | HMDB0004400 | Orotate | HMDB0000226 |
| Abscisic acid | HMDB0036093 | O-Succinyl-Homoserine | HMDB0255868 |
| Acetaminophen | HMDB0001859 | Oxalic acid | HMDB0002329 |
| Acetoacetate | HMDB0304256 | Palatinose | HMDB0256083 |
| Adenine | HMDB0000034 | Pantothenate | HMDB0000210 |
| Adipic acid | HMDB0000448 | P-Coumaric acid 4-O-Sulfate | HMDB0125166 |

| | | | |
|---|---|---|---|
| Agmatine Sulfate | HMDB0001432 | P-Cresol | HMDB0001858 |
| Allantoin | HMDB0000462 | P-Cresol Glucuronide | HMDB0011686 |
| Alpha-Aminobutyric acid | HMDB0000452 | P-Cresol Sulfate | HMDB0011635 |
| Alpha-Hydroxyhippuric acid | HMDB0002404 | Peg200 1 | HMDB0037790 |
| Aminoadipate | HMDB0000510 | Peg200 4 | HMDB0037790 |
| Aminohippuric acid | HMDB0001867 | Peg200 5 | HMDB0037790 |
| Androsterone Glucuronide | HMDB0002829 | Peg200 6 | HMDB0037790 |
| Aniline-2-Sulfonate | HMDB0304940 | Peg200 7 | HMDB0037790 |
| Anisaldehyde | HMDB0029686 | Peg200 8 | HMDB0037790 |
| Anserine | HMDB0000194 | Peg200 9 | HMDB0037790 |
| Anthranilate | HMDB0001123 | Peonidin-3-O-(6-Acetyl)-Glucoside | HMDB0301893 |
| Arabinose | HMDB0000646 | Perillic acid | HMDB0004586 |
| Arabitol | HMDB0000568 | Phenethylamine | HMDB0012275 |
| Ascorbate | HMDB0000044 | Phenylacetylglutamine | HMDB0006344 |
| Aspartate | HMDB0000191 | Phenylethanolamine | HMDB0001065 |
| Asp-Phe | HMDB0000706 | Phloroglucinol | HMDB0013675 |
| Azelate | HMDB0000784 | Phosphoric acid | HMDB0001429 |
| Beta-Alanine | HMDB0000056 | Phosphoserine | HMDB0000272 |
| Biotin | HMDB0000030 | Pimelic acid | HMDB0000857 |
| Butyryl-L-Carnitine | HMDB0002013 | Pipecolate | HMDB0000070 |
| Cadaverine | HMDB0002322 | P-Octopamine | HMDB0004825 |
| Caffeate | HMDB0001964 | Prednisolone | HMDB0014998 |
| Caffeic acid-3-O-Sulfate | HMDB0041706 | Pregnenolone Sulfate | HMDB0000774 |
| Caffeine | HMDB0001847 | Pro-Leu | HMDB0253028 |
| Carbamoyl-Dl-Aspartic acid | HMDB0000828 | Psicose | HMDB0250793 |
| Carnosine | HMDB0000033 | Pterin | HMDB0000802 |
| Cellobiose | HMDB0000055 | Putrescine | HMDB0001414 |
| Cholate | HMDB0000619 | Pyridoxal | HMDB0001545 |
| Cinnamoylglycine | HMDB0011621 | Pyridoxamine | HMDB0001431 |
| Cis-2-Hydroxypenta-2,4-Dienoic acid | | Pyridoxine | HMDB0000239 |
| Cis-4-Hydroxy-D-Proline | HMDB0060460 | Pyrocatechol | HMDB0000957 |
| Cis-Vaccenic acid | HMDB0240219 | Pyroglu-Ile | HMDB0341381 |
| Citrulline | HMDB0000904 | Pyroglutamate | HMDB0000267 |
| Cmpf | HMDB0061112 | Pyroglu-Val | HMDB0094651 |
| Coenzyme Q1 | HMDB0002012 | Pyroglu-Leu | HMDB0341382 |
| Coenzyme Q2 | HMDB0006709 | Quinate | HMDB0003072 |
| Commendamide | | Quinolinate | HMDB0000232 |
| Coniferin | HMDB0013682 | Resorcinol | HMDB0032037 |
| Cortexolone | HMDB0000015 | Resveratrol | HMDB0003747 |
| Corticosterone | HMDB0001547 | Resveratrol 3-O-Glucuronide | HMDB0041782 |
| Cortisol | HMDB0000063 | Retinoate | HMDB0001852 |
| Cortisone | HMDB0002802 | Ribitol | HMDB0000508 |
| Cyclic Amp | HMDB0000058 | Riboflavin | HMDB0000244 |
| Cyclic Gmp | HMDB0001314 | Ribose | HMDB0000283 |
| Cyclic(Ala-Ile) | | Saccharate | HMDB0000663 |
| Cyclic(Ala-Leu) | | S-Adenosylmethionine | HMDB0001185 |
| Cyclic(Glu-Ile) | | Salicylamide | HMDB0015687 |
| Cyclic(Glu-Val) | | Salicylate | HMDB0001895 |

| | | | |
|---|---|---|---|
| Cyclic(Ile-Val) | | Salsolinol | HMDB0042012 |
| Cyclic(Phe-Val) | | Sebacic acid | HMDB0000792 |
| Cyclo(Leucyl-Prolyl) | HMDB0034276 | Serotonin | HMDB0000259 |
| Cyclo(Pro-Val) | HMDB0240493 | Shikimate | HMDB0003070 |
| Cystathionine | HMDB0000099 | Sorbitol | HMDB0000247 |
| Cysteine | HMDB0251515 | S-Sulfocysteine | HMDB0000731 |
| Cytosine | HMDB0000630 | Stachyose | HMDB0003553 |
| D-(-)-Erythrose | HMDB0002649 | Suberate | HMDB0000893 |
| D-(+)Galacturonic acid | HMDB0002545 | Succinate Semialdehyde | HMDB0001259 |
| Decanoyl-L-Carnitine | HMDB0000651 | Succinyladenosine | HMDB0000912 |
| Dehydroascorbate | HMDB0001264 | Syringic acid | HMDB0002085 |
| Dehydrocholic acid | | Syringin | |
| Delta Cehc | HMDB0242106 | Tagatose | HMDB0003418 |
| Deoxycarnitine | HMDB0001161 | Takeda Ketol | |
| Deoxycytidine | HMDB0000014 | Taurochenodeoxycholate | HMDB0000951 |
| Deoxyguanosine | HMDB0000085 | Taurocholic acid | HMDB0000036 |
| Deoxyguanosine-Monophosphate | HMDB0001044 | Taurodeoxycholic acid | HMDB0000896 |
| Deoxyuridine | HMDB0000012 | Tert-Butylhydroquinone | HMDB0032062 |
| Dethiobiotin | HMDB0003581 | Testosterone Glucuronide | HMDB0003193 |
| D-Glucuronolactone | HMDB06355 | Tetrahydrohippuric acid | HMDB0061679 |
| Dihydro Isoferulic acid-3-O-Glucuronide | | Theobromine | HMDB0002825 |
| Dihydro Isoferulic acid-3-O-Sulfate | HMDB0041748 | Theophylline | HMDB0001889 |
| Dihydro-8-Deoxylactucin-15-Glycoside | | Threonic acid | HMDB0000943 |
| Dihydrocaffeic acid-3-O-Glucuronide | HMDB0041720 | Thymidine-Monophosphate | HMDB0001227 |
| Dihydroferulic acid | HMDB0062121 | Thymine | HMDB0000262 |
| Dihydroferulic acid-4-O-Glucuronide | HMDB0041723 | Tiglyl-L-Carnitine | HMDB0002366 |
| Dimethylmaleate | HMDB0036232 | Trans-2-Decenoyl-L-Carnitine | |
| Diphosphoric acid | HMDB0304340 | Trans-3-Hydroxycinnamic acid | HMDB0001713 |
| Dl-3-Indolelactic acid | HMDB0000671 | Trans-Aconitate | HMDB0000958 |
| Dl-Benzylsuccinic acid | HMDB0012127 | Trans-Cinnamaldehyde | HMDB0258628 |
| Dl-Beta-Aminobutyric acid | HMDB0031654 | Trehalose | HMDB0000975 |
| D-Mannosamine | | Tricarballylic acid | HMDB0031193 |
| Dopamine | HMDB0000073 | Trigonelline | HMDB0000875 |
| D-Raffinose | HMDB0003213 | Tryptamine | HMDB0000303 |
| D-Ribose 5-Phosphate | HMDB0001548 | Tyramine | HMDB0000306 |
| D-Ribulose | HMDB0000621 | Umbelliferone | HMDB0029865 |
| D-Xylonic acid | HMDB0059750 | Undecanedioic acid | HMDB0000888 |
| Ectoine | HMDB0240650 | Uracil | HMDB0000300 |
| Epinephrine | HMDB0000068 | Urate | HMDB0000289 |
| Ethylmalonate | HMDB0000622 | Ureidopropionate | HMDB0000026 |
| Ferulate | HMDB0000954 | Uridine | HMDB0000296 |
| Ferulic acid 4-O-Sulfate | HMDB0029200 | Ursodeoxycholic acid | HMDB0000946 |
| Fmnh | HMDB0001142 | Val-Glu | HMDB0029126 |
| Fructose Bisphosphate | HMDB0001058 | Vanillic acid | HMDB0000484 |
| Fucose | HMDB0000174 | Xanthine | HMDB0000292 |
| Galactitol | HMDB0000107 | Xanthurenate | HMDB0000881 |
| Galactose | HMDB0033704 | | |

**Supplementary table S5.** List of functional blocks and their respective compounds, according to the metabolomic technique applied.

| RMN approach (n = 14) | |
|---|---|
| **Functional block** | **Metabolites** |
| Amino acids metabolism | 4-Hydroxyproline |
| | Glycine |
| | Hippuric acid |
| | L-Alanine |
| | L-Glutamic acid |
| | L-Glutamine |
| | L-Histidine |
| | L-Leucine |
| | N-Acetyl-L-Aspartic acid |
| | Sarcosine |
| | Urea |
| CHO metabolism | 4-DEA |
| | 4-DTEA |
| | Alpha-Hydroxyisobutyric acid |
| | Alpha Lactose |
| | D-Galactose |
| | D-Glucose |
| | D-Xylose |
| | L Alanine |
| | Sucrose |
| Energy metabolism | 3-Hydroxyisovaleric acid |
| | Acetic acid |
| | Acetone |
| | Creatine |
| | Creatinine |
| | D-Galactose |
| | Glycine |
| | L-Carnitine |
| | L-Isoleucine |
| | Pyruvic acid |
| Epigenetic modification | Betaine |
| | Sarcosine |
| | Succinic acid |
| Exogenous compound | Benzoic acid |
| | Dimethylamine |
| | Methanol |
| | Propylene Glycol |
| | Trimethylamine |
| Fatty acid metabolism | 3-Methyladipic acid |
| | Acetone |
| | L-Carnitine |
| Metabolic regulation | 1-Methylnicotinamide |
| | Alpha-Hydroxyisobutyric acid |
| | Creatine |
| | Creatinine |
| | Dimethylglycine |
| | Ethanol |
| | Gluconic acid |
| | Hippuric acid |
| | L-Isoleucine |
| | L-Valine |
| | Sucrose |
| Metabolic intermediates | L-Tryptophan |
| | L-Tyrosine |
| | Pyruvic acid |
| | Succinic acid |
| Microbiota metabolism | 3-Indoxylsulfate |
| | 4-Hydroxyproline |
| | Acetic acid |
| | Acetone |
| | Benzoic acid |
| | D-Xylose |
| | Hippuric acid |
| | Methanol |
| | p-Hydroxyphenylacetic acid |
| | Phenylacetylglycine |
| | Propylene Glycol |
| | Trimethylamine |
| | Trimethylamine-N-oxide |

| | |
|---|---|
| | Xanthosine |
| Muscle metabolism | 3-Hydroxyisovaleric acid |
| | Creatine |
| | Creatinine |
| | L-Carnitine |
| | L-Lysine |
| | L-Valine |
| | Sarcosine |
| Neuronal metabolism and neurotransmitters | Betaine |
| | N-Acetyl-L-Aspartic acid |
| | N-Methylnicotinamide |
| | L-Glutamic acid |
| | L-Glutamine |
| | L-Tyrosine |
| | Taurine |
| Protein metabolism | 3-Hydroxyisovaleric acid |
| | L-Alanine |
| | L-Glutamic acid |
| | L-Glutamine 1 |
| | L-Leucine 1 |
| | Oxoglutaric acid |
| | Urea |
| TCA | Cis-Aconitic acid |
| | Citric acid |
| | Fumaric acid |
| | Oxoglutaric acid |
| | Pyruvic acid |
| | Succinic acid |
| Uremic toxins | 3-Indoxylsulfate |
| | Dimethylamine |
| | Ethanol |
| | Methylguanidine |
| | Propylene glycol |
| | Trimethylamine |
| | Urea |
| **LC-MS approach (n = 66)** | |
| **Functional block** | **Metabolites** |
| Dipeptides | Asp-Phe |
| | Cyclic Ala-Ile |
| | Cyclic Ala-Leu |
| | Cyclic Glu-Ile |
| | Cyclic Glu-Val |
| | Cyclic Phe-Val |
| | Cyclo Leucyl-Prolyl |
| | Cystathionine |
| | Glu-Val |
| | Gly-Gly |
| | Gly-Pro |
| | H-Leu-Ile-Oh |
| | H-Leu-Pro-Oh |
| | H-Leu-Tyr-Oh |
| | Pro-Leu |
| | Pyroglu-Ile |
| | Pyroglu-Val |
| | Pyruglu-Leu |
| | Val-Glu |
| Amino acids metabolism | Cadaverine |
| | 4-Hydroxyproline |
| | Cysteine |
| | Ectoine |
| | Glutarate |
| | Gly-Gly |
| | Glycine |
| | Hippuric acid |
| | Imidazolepropionic Acid |
| | L-Arginine |
| | L-Asparagine |
| | L-Glutamic Acid |
| | L-Glutamine |
| | L-Histidine |
| | L-Ornithine |
| | L-Phenylalanine |
| | L-Serine |
| | L-Threo-3-Phenylserine |
| | L-Threonine |

| | |
|---|---|
| | N-Acetylasparagine |
| | N-Acetylaspartate |
| | N-Acetylcysteine |
| | N-Acetylphenylalanine |
| | N-Acetylputrescine |
| | N-Methyl-DL-Alanine |
| | N-Methylaspartate |
| | Phenylacetylglutamine |
| | Pimelic Acid |
| | Pipecolate |
| | Putrescine |
| | Pyroglutamate |
| | 1,3-Diaminopropane |
| | 2-Aminoisobutyrate |
| | 2-Oxo-4-Phenyl Butyric Acid |
| | 2-Oxoadipate |
| | 2-Oxobutanoic Acid |
| | 3-Methylhistamine |
| | 4-Acetamidobutanoate |
| | 5-Hydroxymethyl-2-Furancarboxylic Acid |
| Antiinflammatory | Azelate |
| | Caffeine |
| | Cortisone |
| | Glu-Val |
| | Methylguanidine |
| | N-Acetyltryptophan |
| | Prednisolone |
| | Resveratrol |
| | Resveratrol-3-O-Glucuronide |
| | Salicylamide |
| | Stachyose |
| | Syringic Acid |
| | Syringin |
| | 2-Hydroxybenzyl alcohol |
| | 3-Hydroxybenzyl alcohol |
| | Acetaminophen |
| | 3-Hydroxybenzaldehyde |
| Antioxidant | Aamu |
| | Ascorbate |
| | Caffeic Acid 3-O-Sulfate |
| | Citrulline |
| | Coenzyme Q1 |
| | Coenzyme Q2 |
| | Cyclo Leucyl-Prolyl |
| | Cysteine |
| | D-Glucuronolactone |
| | Dehydroascorbate |
| | Delta Cehc |
| | Dihydro-8-Deoxylactucin-15 Glycoside |
| | Dihydro Isoferulic acid 3-O-Glucuronide |
| | Dihydro Isoferulic acid 3-O-Sulfate |
| | Dihydrocaffeic acid 3-O-Glucuronide |
| | Dihydroferulic acid |
| | Dihydroferulic acid 4-O-Glucuronide |
| | Ferulate |
| | Ferulic acid 4-O-Sulfate |
| | Gallic acid |
| | Hydroxyphenyllactate |
| | Hydroxytyrosol |
| | Hypotaurine |
| | Inosine |
| | Isoferulic acid 3-O-Glucuronide |
| | N-Acetylcysteine |
| | N-Acetylmethionine |
| | N-Acetyltryptophan |
| | p-Coumaric acid 4-O-Sulfate |
| | Resveratrol |
| | Resveratrol 3-O-Glucuronide |
| | Syringic acid |
| | Taurine |
| | Tert-Butylhydroquinone |
| | Trans-3-Hydroxycinnamic acid |
| | Umbelliferone |
| | Urate |
| | 1,7-Dimethyluric acid |
| | 1-Methyl Pyrogallol 3-O-Sulfate |

| | |
|---|---|
| | 1-Methyluric acid<br>2,4-Dihydroxycinnamic acid<br>3,4-Dihydroxybenzaldehyde<br>3,4-Dihydroxybenzoate<br>3-Ethoxy-4-Hydroxybenzaldehyde<br>3-Hydroxyanthranilate<br>3-Hydroxybenzyl alcohol<br>3-Hydroxyphenylacetate<br>3-Indolepropionic acid<br>3-Sulfinoalanine<br>4(4-Hydroxyphenyl)Butan-2-One<br>4-Methylcatechol Monosulfate<br>3,4-Dihydroxyhydrocinnamic acid |
| Arginine metabolism | Agmatine Sulfate<br>L-Arginine<br>L-Ornithine<br>N-Acetylputrescine<br>4-Guanidinobutanoate |
| Bile acid metabolism | Cholate<br>Glycochenodeoxycholate<br>Glycocholate<br>Glycodeoxycholic acid<br>Glycohyocholic_acid<br>Nordeoxycholic acid<br>Sodium Taurochenodeoxycholate<br>Takeda Ketol<br>Taurocholic acid<br>Taurodeoxycholic acid<br>Ursodeoxycholic acid<br>12-Ketochenodeoxycholic acid<br>3-Hydroxy-7,12-Diketocholanoic acid<br>3-Hydroxy-7-Oxo-5-Cholanicacid<br>5B-Cholanic acid 3A-6A-7A-Triol<br>7-Ketodeoxycholic acid |
| Branched chain amino acids metabolism | Isovaleroylglycine<br>Isovaleryl L-Carnitine<br>N-Acetyl-DL-Valine<br>3-Methyl-2-Oxovalerate<br>3-Methylcrotonyl-L-Carnitine<br>Isoleucine<br>L-Leucine<br>Methyl-Succinic acid<br>N-Acetylleucine<br>Norvaline<br>Valine<br>(E)-3-Methylglutaconic acid<br>2-Hydroxy-3-Methylbutyric acid<br>2-Hydroxyisocaproic acid<br>2-Isopropylmalic acid<br>3-Isopropylmalic acid<br>3-Methylglutaric acid<br>4-Methyl-2-Oxovaleric acid |
| Caffeine metabolism | Caffeic acid 3-O-Sulfate<br>Dihydrocaffeic acid 3-O-Glucuronide<br>1,7-Dimethyluric Acid<br>7-Methylxanthine |
| Catecholamine metabolism | Homovanillate<br>Homovanillic acid Sulfate<br>(4R)-5-(3,4-Dihydroxyphenyl)-Gamma-Valerolactone-4'-O-Sulfate<br>(R)-(-)-Phenylephrine<br>3,4-Dihydroxymandelate<br>3-Hydroxymandelic acid<br>3-Methoxycatechol<br>Ascorbate<br>Dopamine<br>Epinephrine<br>L-Phenylalanine<br>L-Serine<br>N,N-Dimethyl-1,4-Phenylenediamine<br>Normetanephrine<br>p-Octopamine<br>3,4-Dihydroxy-L-Phenylalanine<br>3,4-Dihydroxyphenylacetate<br>3,4-Dihydroxyphenylglycol<br>3-Methoxytyramine |

| | |
|---|---|
| | 3-Methoxytyrosine |
| | 7,8-Dihydro-L-Biopterin |
| Cell growth and proliferation | Cadaverine |
| | D-Mannosamine |
| | Diphosphoric acid |
| | Histidinol |
| | N-Acetylputrescine |
| | N6-(Delta2-Isopentenyl)-Adenine |
| | Norvaline |
| | Putrescine |
| | Trans-Cinnamaldehyde |
| | 2’-O-Methyladenosine |
| | 2-Methoxy-4-Vinylphenol |
| | Ectoine |
| | Retinoate |
| | 16-0 Lysopa |
| Cell signaling | Commendamide |
| | Cyclic Amp |
| | Cyclic Gmp |
| | Myoinositol |
| | Phosphoserine |
| | Serotonin |
| | 16-0 Lysopa |
| | 2-Hydroxypyridine |
| | 3-Methoxycatechol |
| | 7-Methylxanthine |
| Cell transduction and communication | Cyclic Amp |
| | Cyclic Gmp |
| | N-Acetylgalactosamine |
| | N-Acetylglucosamine |
| CHO metabolism | Alpha-D-Glucose |
| | Arabinose |
| | Arabitol |
| | D-(-)-Erythrose |
| | D-Glucuronolactone |
| | D-Mannosamine |
| | D-Raffinose |
| | D-Ribose-5-Phosphate |
| | D-Xylonic acid |
| | Fructose Bisphosphate |
| | Fucose |
| | Galactose |
| | Gluconolactone |
| | Glucose-1-Phosphate |
| | Glucose-6-Phosphate |
| | Glycerol-3-Phosphate |
| | Lactose |
| | Maltose |
| | Maltotriose |
| | Mannitol |
| | Melibiose |
| | N-Acetyl-DL-Valine |
| | Ribitol |
| | Ribose |
| | Saccharate |
| | Sucrose |
| | Threonic Acid |
| | Xylose |
| | Abscisic Acid |
| Cholesterol metabolism | Cholate |
| | Glycochenodeoxycholate |
| | Glycocholate |
| | Glycodeoxycholic acid |
| | Mevalolactone |
| | Nordeoxycholic acid |
| | Sodium Taurochenodeoxycholate |
| | Takeda Ketol |
| | Taurocholic acid |
| | Taurodeoxycholic acid |
| | Ursodeoxycholic_acid |
| | 12-Ketochenodeoxycholic acid |
| | 3-Hydroxy-7,12-Diketocholanoic acid |
| Collagen catabolism | Gly-Pro |
| | Cis-4-Hydroxy-D-Proline |
| | L-Threonine |

| | |
|---|---|
| | Trans-4-Hydroxy-L-Proline<br>5-Hydroxylysine |
| Cystein and homocystein metabolism | Betaine<br>Cystathionine<br>Homocysteine<br>L-Methionine<br>N-Acetylmethionine<br>O-Succinyl Homoserine<br>S-Adenosylmethionine<br>2-Oxobutanoic acid<br>3-Sulfinoalanine |
| Detoxication | D-(+)Galacturonic acid<br>D-Glucuronolactone<br>Galacturonate<br>Glucuronate<br>Isoferulic acid 3-O-Glucuronide<br>Methyl-Glucuronide<br>Saccharate<br>Tetrahydrohippuric acid<br>2-Methylhippuric acid |
| Drug | Acetaminophen<br>Prednisolone<br>Resorcinol<br>Salicylamide |
| Energy metabolism | Acetoacetate<br>Aniline-2-Sulfonate<br>Creatine<br>Creatinine<br>Deoxycarnitine<br>Diphosphoric acid<br>Galactose<br>Glycine<br>Guanidinoacetate<br>Isoleucine<br>L-Carnitine<br>N,N,N-Trimethyllysine<br>O-Acetyl-L-Carnitine<br>Octenoylcarnitine<br>Pyruvic acid<br>Trans-Cinnamaldehyde<br>(S)-3-Hydroxybutyric acid |
| Epigenetic modification | L Methionine<br>N-Acetylglucosamine<br>N-Alpha Acetyllysine<br>N2-Methylguanosine<br>S-Adenosylmethionine<br>Succinic Acid<br>5-Methylcytosine<br>7-Methylguanine |
| Fatty acid B-oxidation | Acetoacetate<br>Decanoyl-L-Carnitine<br>Deoxycarnitine<br>Ethylmalonate<br>Hexanoyl-L-Carnitine<br>L-Carnitine<br>Nonanoylcarnitine<br>Octanoyl-L-Carnitine<br>Octenoylcarnitine<br>(S)-3-Hydroxybutyric acid<br>3-Hydroxybutanoate |
| Fatty acid metabolism | Azelate<br>Cis-Vaccenic acid<br>Heptylmalonic acid<br>Hexanoic acid<br>Hexanoylglycine<br>Octanoic acid Glucuronide<br>Pimelic acid<br>Suberate<br>Undecanedioic acid<br>2,2-Dimethylglutaric acid<br>2-Ethylsuberic acid<br>2-Hydroxydodecanoic acid<br>2-Methylhippuric acid<br>3,3-Dimethylglutaric acid<br>3-Methyladipic acid |

| | |
|---|---|
| | Linoleic acid |
| Ganglioside metabolism | Fucose |
| | N-Acetylmannosamine |
| | N-Acetylneuraminate |
| Gene expression | D-Ribose-5-Phosphate |
| | Dethiobiotin |
| | Isonicotinic acid N-Oxide |
| | L-Methionine |
| | N-Alpha-Acetyllysine |
| | N,N,N-Trimethyllysine |
| | Retinoate |
| | 1-Methyladenosine |
| | 2'-O-Methyladenosine |
| | 2'-O-Methylinosine |
| | 2-Acetamido-2-Deoxy-Beta-D-Glucosylamine |
| | 7-Methylguanine |
| General metabolism | Adenine |
| | Biotin |
| | Carnosine |
| | Cytosine |
| | Dethiobiotin |
| | Fmnh |
| | Guanine |
| | Pantothenate |
| | Riboflavin |
| Gut function | D-Raffinose |
| | Sucrose |
| | 2-Hydroxyisocaproic acid |
| Immune function | Abscisic acid |
| | Histamine |
| | Indole-3-Carboxaldehyde |
| | Isonicotinic acid N-Oxide |
| | N-Formyl-L-Methionine |
| | 1-Methyladenosine |
| | 3-Phenyllactic acid |
| Inflammation | L-Histidine |
| | Linoleic acid |
| | 14-0 Lysope |
| | 2,2-Dimethylglutaric acid |
| | 2,6-Dihydroxybenzoic acid |
| | 3-Methylhistamine |
| | 3-Nitro-L-Tyrosine |
| | 4-Aminobenzoate |
| | 5-Aminolevulinate |
| | 5-Aminopentanoate |
| | 5-Hydroxymethyl-2-Furancarboxylic acid |
| | 5-Methoxyindoleacetic acid |
| Kidney function | Aminohippuric acid |
| | Cmpf |
| | Methylguanidine |
| | N-Acetylalanine |
| | N-Acetylserine |
| | N-Methyl-2-Pyridone-5-Carboxamide |
| | Oxalic acid |
| | Phosphoric acid |
| | Trigonelline |
| | D-(+)Galacturonic acid |
| | Galacturonate |
| | Histidinol |
| | Glyoxylic Acid |
| Lipid metabolism | Acetoacetate |
| | Nonanoylcarnitine |
| | Trans-2-Decenoyl-L-Carnitine |
| | (S)-3-Hydroxybutyric acid |
| | 10-Hydroxyoctadecanoic acid |
| | 3-Hydroxybutanoate |
| Metabolic activation | Betaine |
| | Cortisol |
| | N-Carbamyl-L-Glutamic acid |
| | O-Acetyl-L-Carnitine |
| | Pantothenate |
| Metabolic regulation | Adipic Acid |
| | Aminoadipate |
| | Aminohippuric acid |
| | Arabinose |

Arabitol
Butyryl-L-Carnitine
Cmpf
Creatine
Creatinine
Decanoyl-L-Carnitine
Ethylmalonate
Fucose
Galactitol
Gluconate
Glutarate
Guanidinosuccinate
Hexanoyl-L-Carnitine
Hexanoylglycine
Homogentisic acid
Hydroxykynurenine
Hydroxyphenyllactate
Imidazolepropionic acid
Indole-3-Acryloylglycine
Indole-3-Ethanol
Indoleacetyl Glutamine
Isoleucine
Isovaleroylglycine
Isovaleryl-L-Carnitine
Kynurenic acid
L-Ornithine
Maltose
Mannitol
N-Acetylalanine
N-Acetylglycine
N-Acetylphenylalanine
Octanoyl-L-Carnitine
Oxalic acid
Phosphoric acid
Phosphoserine
S-Sulfocysteine
Saccharate
Sebacic acid
Sorbitol
Succinyladenosine
Sucrose
Tagatose
Threonic acid
Tigloyl-L-carnitine
Trans-2-Decenoyl-L-Carnitine
Trans-Aconitate
Trigonelline
Urate
Valine
(E)-3-Methylglutaconic acid
1,5-Anhydro-D-Sorbitol
2-Acetamido-2-Deoxy-Beta-D-Glucosylamine
2-Aminoisobutyrate
2-Hydroxy-3-Methylbutyric acid
2-Hydroxyisocaproic acid
3-Hydroxymethylglutarate
3-Methyl-2-Oxovalerate
3-Methylcrotonyl-L-Carnitine
3-Methylglutaric acid
4-Guanidinobutanoate
4-Methyl-2-Oxovaleric acid
5-Methylthioribose
Psicose
Carbamoyl-Dl-Aspartic acid
Nicotinic acid
N(6)-Carboxymethyllysine
Hippurate
Glycohyocholic acid
3-Indole Carboxylic acid Glucuronide
N,N-Dimethylguanosine
Phenylethanolamine
Glyoxylic acid
Indole-3-Carboxaldehyde
Methylglutarylcarnitine
2-Ethylsuberic acid
10-Hydroxyoctadecanoic acid

| | |
|---|---|
| Metabolic intermediates | L-Tyrosine<br>Nicotinamide Mononucleotide<br>Pyridoxal<br>Pyridoxine<br>Pyruvic acid<br>Succinic acid<br>Thymine<br>Tryptophan<br>Uracil |
| Microbiota metabolism | 3-Methylindole<br>Arabinose<br>Cellobiose<br>Cinnamoylglycine<br>Cis-2-Hydroxypenta-2,4-Dienoic acid<br>Cis-4-Hydroxy-D-Proline<br>Commendamide<br>Cyclo Pro-Val<br>Dl-Benzylsuccinic acid<br>Glycerate<br>Glycocholate<br>Hippurate<br>Hydroxyphenyllactate<br>Indole<br>Indole-3-Acetate<br>Indole-3-Ethanol<br>Indoleacetyl Glutamine<br>Indoxyl-Beta-D-Glucuronide<br>Indoxyl Sulfate<br>Ll-2,6-Diaminoheptanedioate<br>Lyxose<br>m-Cresol Sulfate<br>Melibiose<br>Methanesulfonic acid<br>N-(2-Phenylacetyl)Glycine<br>N-Formyl-L-Methionine<br>N-Methylglutamate<br>N6-(Delta2-Isopentenyl)-Adenine<br>O-Acetylserine<br>O-Hydroxyhippuric acid<br>p-Cresol Glucuronide<br>p-Cresol Sulfate<br>Phenethylamine<br>Phenylacetylglutamine<br>Pipecolate<br>Ribitol<br>Salicylate<br>Shikimate<br>Threonic acid<br>Tricarballylic acid<br>Trimethylamine-N-Oxide<br>Tryptamine<br>Tyramine<br>Ursodeoxycholic acid<br>12-Ketochenodeoxycholic acid<br>2,6-Dihydroxybenzoic acid<br>2-Hydroxycaproic acid<br>2-Hydroxydodecanoic acid<br>2-Methoxyresorcinol<br>3-(2,4-Dihydroxyphenyl)Propionic acid<br>3-(3-Hydroxyphenyl)Propionic_acid<br>3,4-Dihydroxyhydrocinnamic acid<br>3-Amino-4-Hydroxybenzoate<br>3-Hydroxy-7,12-Diketocholanoic acid<br>3-Hydroxyanthranilate<br>3-Hydroxybenzoate<br>3-Indole Carboxylic acid Glucuronide<br>3-Indolepropionic acid<br>3-Methyl-2-Oxindole<br>3-Phenyllactic acid<br>4-Aminobenzoate<br>4-Hydroxybenzaldehyde<br>4-Hydroxybenzoic acid<br>4-Hydroxyhippuric acid<br>5-Hydroxyindole<br>5-Hydroxyindole-3-Acetic acid<br>Xanthosine |

| | |
|---|---|
| | Xylose |
| | Indole-3-Acetic acid Glucuronide |
| | Glycohyocholic acid |
| | 4-Ethylphenyl Sulfate Ammonium |
| | Indole-3-Carboxaldehyde |
| | m-Hydroxyhippuric acid |
| | Dl-3-Indolelactic acid |
| | 3-Hydroxyphenylvaleric acid |
| | D-Ribulose |
| | p-Cresol |
| | 3-(3,5-Dihydroxyphenyl)Propanoic acid |
| | 3-(3-Hydroxyphenyl)-3-Hydroxypropionic acid |
| | 10-Hydroxyoctadecanoic acid |
| | 2-Indolecarboxylic acid |
| | 7-Ketodeoxycholic acid |
| Mitochondrial function | Adipic acid |
| | Aspartate |
| | Beta-Alanine |
| | Butyryl-L-Carnitine |
| | Coenzyme Q2 |
| | Decanoyl-L-Carnitine |
| | Ethylmalonate |
| | Glutarate |
| | Hexanoyl-L-Carnitine |
| | Hexanoylglycine |
| | Isovaleroylglycine |
| | Isovaleryl-L-Carnitine |
| | Methylglutarylcarnitine |
| | N-Formyl-L-Methionine |
| | Nonanoylcarnitine |
| | O-Acetyl-L-Carnitine |
| | Octanoic acid Glucuronide |
| | Octanoyl-L-Carnitine |
| | Octenoylcarnitine |
| | Tigloyl-L-carnitine |
| | Trans-2-Decenoyl-L-Carnitine |
| | Ureidopropionate |
| | (E)-3-Methylglutaconic acid |
| | 2.Aminoisobutyrate |
| | 2-Oxoadipate |
| | 2-Oxobutanoic acid |
| | 3-Hydroxymethylglutarate |
| | 3-Methylcrotonyl-L-Carnitine |
| | 3-Methylglutaric acid |
| Multiple disorders | Kynurenine |
| | Myoinositol |
| | Normetanephrine |
| | Tryptophan |
| Muscle metabolism | Aniline 2-Sulfonate |
| | Anserine |
| | Beta-Alanine |
| | Butyryl-L-Carnitine |
| | Cadaverine |
| | Citrulline |
| | Creatine |
| | Creatinine |
| | L-Carnitine |
| | L-Leucine |
| | L-Lysine |
| | Norvaline |
| | Valine |
| Neurological dysfunction | Azelate |
| | p-Cresol |
| | O-Methylsinapic acid |
| | N2-Acetylaminoadipic acid |
| Neuronal metabolism and neurotransmitters | Dl-Beta-Aminobutyric acid |
| | L-Threonine |
| | N-Acetylserotonin |
| | Tryptamine |
| | 3,4-Dihydroxyphenylacetate |
| | 3,4-Dihydroxyphenylglycol |
| | Anthranilate |
| | Betaine |
| | Gly-Pro |
| | Inosine |

| | |
|---|---|
| | N-Acetylaspartate<br>N-Acetylglutamate<br>4-Acetamidobutanoate<br>4-Guanidinobutanoate<br>4-Hydroxy-L-Phenylglycine<br>4-Imidazoleacetate<br>5-Hydroxytryptophan |
| Neuroprotection | Agmatine Sulfate<br>Guanine<br>Guanosine<br>Orotate<br>3-Indolepropionic acid<br>4-Methylcatechol<br>Xanthine |
| Neurotoxicity | Beta-Alanine<br>Homocysteine<br>Kynurenine<br>Quinolinate<br>Succinate Semialdehyde<br>2-Quinolinecarboxylate<br>3-(3-Hydroxyphenyl)-3-Hydroxypropionic acid<br>3-Methyl-2-Oxovalerate<br>4-Ethylphenyl Sulfate Ammonium<br>4-Methyl-2-Oxovaleric Acid<br>Hydroxykynurenine |
| Neurotransmitter | Aspartate<br>Dopamine<br>Epinephrine<br>Gamma Aminobutyrate<br>Gamma Hydroxybutyric acid<br>Histamine<br>Homovanillate<br>Hypotaurine<br>L-Glutamic acid<br>L-Glutamine<br>L-Tyrosine<br>N-Acetyl-L-Tyrosine<br>N-Methylaspartate<br>Normetanephrine<br>p-Octopamine<br>Phenethylamine<br>Pyroglutamate<br>Salsolinol<br>Serotonin<br>Taurine<br>Tyramine<br>2-Quinolinecarboxylate<br>3,4-Dihydroxy-L-Phenylalanine<br>Xanthurenate |
| NO metabolism | Citrulline<br>L-Arginine<br>(R)-(-)-Phenylephrine<br>7,8-Dihydro-L-Biopterin |
| Nucleic acid metabolism | Ribose<br>Thymidine Monophosphate<br>Uracil<br>Uridine<br>3,4-Dihydroxybenzenesulfonic acid |
| Nucleotide metabolism | Deoxycytidine<br>Deoxyguanosine<br>Deoxyguanosine Monophosphate<br>Deoxyuridine<br>Ribose<br>5’-Deoxyadenosine |
| Oxidative stress | Allantoin<br>Aminoadipate<br>Glycerate<br>Hydroxykynurenine<br>L-Gulonolactone<br>N-(2-Phenylacetyl)Glycine<br>Nicotinamide Mononucleotide<br>Ophthalmate<br>Succinate Semialdehyde<br>1-Methyluric Acid<br>14-0 Lysope |

| | |
|---|---|
| | X3 Nitro L Tyrosine<br>5’-Deoxyadenosine<br>Gluconolactone<br>Syringin |
| Phenylalanine metabolism | L-Threo-3-Phenylserine<br>Phenethylamine<br>Salicylate<br>X2 Oxo 4 Phenyl Butyric Acid<br>X5 Hydroxymethyl 2 Furancarboxylic Acid |
| Phospholipid metabolism | Choline<br>N-(2-Phenylacetyl)Glycine<br>N-Acetylmannosamine<br>N-Acetylneuraminate<br>Uridine<br>14-0 Lysope<br>16-0 Lysopa |
| Post-translational regulation | N-Acetylalanine<br>N-Acetylserine<br>2-Acetamido-2-Deoxy-Beta-D-Glucosylamine |
| Prebiotic | Meso-Tartrate<br>Stachyose<br>Tagatose<br>Trehalose |
| Protein metabolism | Aspartate<br>Cysteine<br>L-Asparagine<br>L-Glutamic acid<br>L-Glutamine<br>L-Leucine<br>Oxoglutarate<br>5-Hydroxylysine<br>Cyclic Glu-Ile<br>Cyclic Ile-Val<br>Cyclic Phe-Val<br>H-Leu-Ile-OH<br>N,N,N-Trimethyllysine<br>Pyroglu-Ile<br>Val-Glu<br>Glu-Val<br>Glucosamine |
| Purine metabolism | Adenine<br>Deoxyguanosine<br>Deoxyguanosine Monophosphate<br>Guanine<br>Guanosine<br>Inosine<br>Succinyladenosine<br>Urate<br>1-Methyladenosine<br>1-Methyluric acid<br>2’-O-Methylinosine<br>3-Methyladenine<br>4-Imidazoleacetate<br>7-Methylxanthine<br>Xanthine<br>Xanthosine |
| Pyrimidine metabolism | Isonicotinic acid N-Oxide<br>Carbamoyl-Dl-Aspartic acid<br>Cytosine<br>Deoxycytidine<br>Deoxyuridine<br>Orotate<br>Pyridoxamine<br>Thymidine Monophosphate<br>Thymine<br>Uracil<br>Ureidopropionate |
| Redox status | Alpha-Aminobutyric acid<br>Carnosine<br>L-Histidine<br>Pyridoxamine<br>Ureidopropionate<br>1,7-Dimethyluric Acid<br>1-Methyl-L-Histidine<br>3-Methyladenine |

| | |
|---|---|
| | 4-Hydroxybenzaldehyde |
| Steroid metabolism | Androsterone Glucuronide<br>Cortexolone<br>Corticosterone<br>Cortisol<br>Cortisone<br>Indole-3-Ethanol<br>Mevalolactone<br>Nordeoxycholic acid<br>Pregnenolone Sulfate<br>Testosterone Glucuronide<br>Tetrahydrocortisol<br>17A-Hydroxypregnenolone<br>17A-Hydroxyprogesterone<br>21-Deoxycortisol |
| Stress response | Carnosine<br>L-Lysine<br>Pyroglutamate<br>4-Hydroxyproline<br>1-Methyl-L-Histidine |
| TCA | Cis-Aconitate<br>Citrate<br>Fumarate<br>Malate<br>Maleamate<br>Maleate<br>Malonate<br>Oxoglutarate<br>Pyruvic acid<br>Succinic acid<br>2-Methylglutarate |
| Tryptophan metabolism | Anthranilate<br>DL-3-Indolelactic acid<br>Hydroxykynurenine<br>Indole<br>Indole-3-Acetate<br>Indole-3-Acryloylglycine<br>Indoleacetyl Glutamine<br>Indoxyl Sulfate<br>Kynurenic acid<br>Kynurenine<br>N-Acetyltryptophan<br>Quinolinate<br>Tryptamine<br>Tryptophan<br>2,6-Quinolinediol<br>2-Aminophenol<br>2-Quinolinecarboxylate<br>3-Hydroxyanthranilate<br>3-Indole-Carboxylic acid Glucuronide<br>3-Methyl-2-Oxindole<br>3-Methylindole<br>5-Hydroxyindole<br>5-Hydroxyindole-3-Acetic acid<br>5-Hydroxytryptophan<br>5-Methoxyindoleacetic acid<br>Xanthurenate<br>N-Acetylserotonin |
| Tyrosine metabolism | Dopamine<br>Homovanillate<br>Hydroxyphenyllactate<br>L-Tyrosine<br>p-Cresol Glucuronide<br>p-Cresol Sulfate<br>Tyramine<br>3,4-Dihydroxy-L-Phenylalanine<br>3,4-Dihydroxyphenylacetate<br>3,4-Dihydroxyphenylglycol<br>3-Hydroxyphenylacetate<br>3-Methoxytyramine<br>3-Methoxytyrosine<br>7,8-Dihydro-L-Biopterin |
| Unknown | Isodehydracetic acid<br>L-Beta-Homoisoleucine Hydrochloride<br>L-Beta-Homomethionine Hcl |

| | |
|---|---|
| | L-Beta-Homophenylalanine Hydrochloride<br>N-Phenylacetyl-Methionine<br>2-Hydroxy-4-Phenylbutyric acid<br>3-(3,4-Dihydroxyphenyl)Lactate<br>3,4-Dihydroxyphenylvaleric acid<br>4-Aminophenyl Hydrogen Sulfate<br>4-Hydroxy-3-Methylbenzoic acid<br>4-Quinolinecarboxylic acid<br>7-(1-Carboxyethyl)Lysine |
| Urea cycle | Guanidinoacetate<br>N-Acetylglutamate<br>N-Carbamyl-L-Glutamic acid<br>Orotate |
| Uremic toxins | Cmpf<br>Indole-3-Acetic acid Glucuronide<br>Indoxyl Beta-D-Glucuronide<br>4-Ethylphenyl Sulfate Ammonium<br>Anthranilate<br>Guanidinosuccinate<br>Indole-3-Acetate<br>Indoxyl Sulfate<br>Methylguanidine<br>N-Methyl-2-Pyridone-5-Carboxamide<br>p-Cresol<br>p-Cresol Glucuronide<br>p-Cresol Sulfate<br>5-Hydroxyindole<br>5-Hydroxyindole-3-Acetic acid |
| Vascular health | Dimethylmaleate<br>Histamine<br>Homocysteine<br>L-Lysine<br>N(6)-Carboxymethyllysine<br>N-Acetylmethionine<br>Nicotinamide Mononucleotide<br>Tetrahydrocortisol<br>Trimethylamine-N-Oxide<br>Undecanedioic acid<br>3,4-Dihydroxybenzaldehyde<br>3-Hydroxybenzaldehyde<br>2’-O-Methyladenosine<br>Tyramine<br>Phenylethanolamine<br>Caffeate<br>Caffeine<br>Theobromine<br>Theophylline<br>2’-O-Methylinosine |
| Vitamin metabolism | Ascorbate<br>Biotin<br>Choline<br>Cystathionine<br>Delta-Cehc<br>Dethiobiotin<br>L-Gulonolactone<br>Lumichrome<br>Pantothenate<br>Pimelic acid<br>Riboflavin<br>4-Pyridoxate<br>Nicotinic acid<br>Pyridoxine<br>Fmnh<br>Methylmalonic acid<br>Retinoate<br>Phosphoric acid |
| w-oxidation | Omega Hydroxydodecanoate<br>Pimelic acid<br>Sebacic acid<br>Suberate<br>Undecanedioic acid<br>3-Methyladipic acid |
| Xenobiotics | Abscisic Acid<br>Anisaldehyde<br>Cinnamoylglycine |

Cis-2-Hydroxypenta-2,4-Dienoic acid
Cyclo Pro-Val
Dehydrocholic acid
Dihydro-8-Deoxylactucin-15-Glycoside
Dihydro-Isoferulic acid 3-O-Glucuronide
Dihydro-Isoferulic acid 3-O-Sulfate
Dihydrocaffeic acid 3-O-Glucuronide
Dihydroferulic acid
Dihydroferulic acid 4-O-Glucuronide
Ferulic acid 4-O-Sulfate
H-Leu-Pro-OH
Hinokitiol
Isoferulic acid 3-O-Glucuronide
p-Coumaric acid 4-O-Sulfate
Perillic acid
Syringin
2-Methoxy-4-Vinylphenol
3-(3,4,5-Trimethoxyphenyl)Propionic acid
3-(3-Hydroxyphenyl)Propionic acid
3-Phenyllactic acid
4-(4-Hydroxyphenyl)Butan-2-One
Acetaminophen
Alpha Hydroxyhippuric acid
Caffeate
Caffeine
Ferulate
Galactitol
Gallic acid
L-Methionine Sulfone
Mesoxalate
Methyl Galactoside
Mono-Methyl Adipate
O-Acetylserine
O-Methylsinapic acid
Palatinose
Peg200_1
Peg200_4
Peg200_5
Peg200_6
Peg200_7
Peg200_8
Peg200_9
Peonidin-3-O-(6-Acetyl)-Glucoside
Pterin
Pyrocatechol
Quinate
Resorcinol
Resveratrol
Resveratrol 3-O-Glucuronide
Salicylamide
Salicylate
Shikimate
Tert-Butylhydroquinone
Theobromine
Theophylline
Trans-3-Hydroxycinnamic acid
Trans-Cinnamaldehyde
Vanillic acid
(-)-Cotinine
1-Methyl Pyrogallol 3-O-Sulfate
1-Methyltryptophan
2',4'-Dihydroxyacetophenone
2,3-Diaminopropionate
2,4,6-Trihydroxybenzoic acid
2,4-Dihydroxybenzaldehyde
2,4-Dihydroxycinnamic acid
2,6-Dihydroxybenzoic acid
2-Hydroxy-4-(Methylthio)Butanoate
3-(2-Hydroxyphenyl)Propanoate
3,4-Dihydroxybenzaldehyde
3,4-Dihydroxybenzoate
3,5-Dihydroxybenzoic Acid
3-Ethoxy-4-Hydroxybenzaldehyde
3-Hydroxybenzaldehyde
3-Hydroxyphenylvaleric Acid
4-Methylcatechol

| |
|---|
| 4-Methylcatechol Monosulfate |
| Asp-Phe |
| Aamu |
| O-Cresol Sulfate |
| 3,4-Dihydroxyhydrocinnamic acid |
| 3-Methylhippuric acid |
| 4-Acetamidobenzoic acid |
| 4-Methylhippuric acid |
| 3-Hydroxybenzyl Alcohol |
| Homoveratric acid |
| Hydroxytyrosol |
| Coniferin |
| Umbelliferone |
| D-Raffinose |
| Syringic acid |
