# Supplementary figures and images for: Neonatal Urine Metabolic Signature Reflects Multisystemic Adaptations Linked to Preterm Birth
Source: Int J Mol Sci. 2025 Sep 14;26(18):8953. doi: 10.3390/ijms26188953 (PMC12469547; doi:10.3390/ijms26188953)

Figure S1

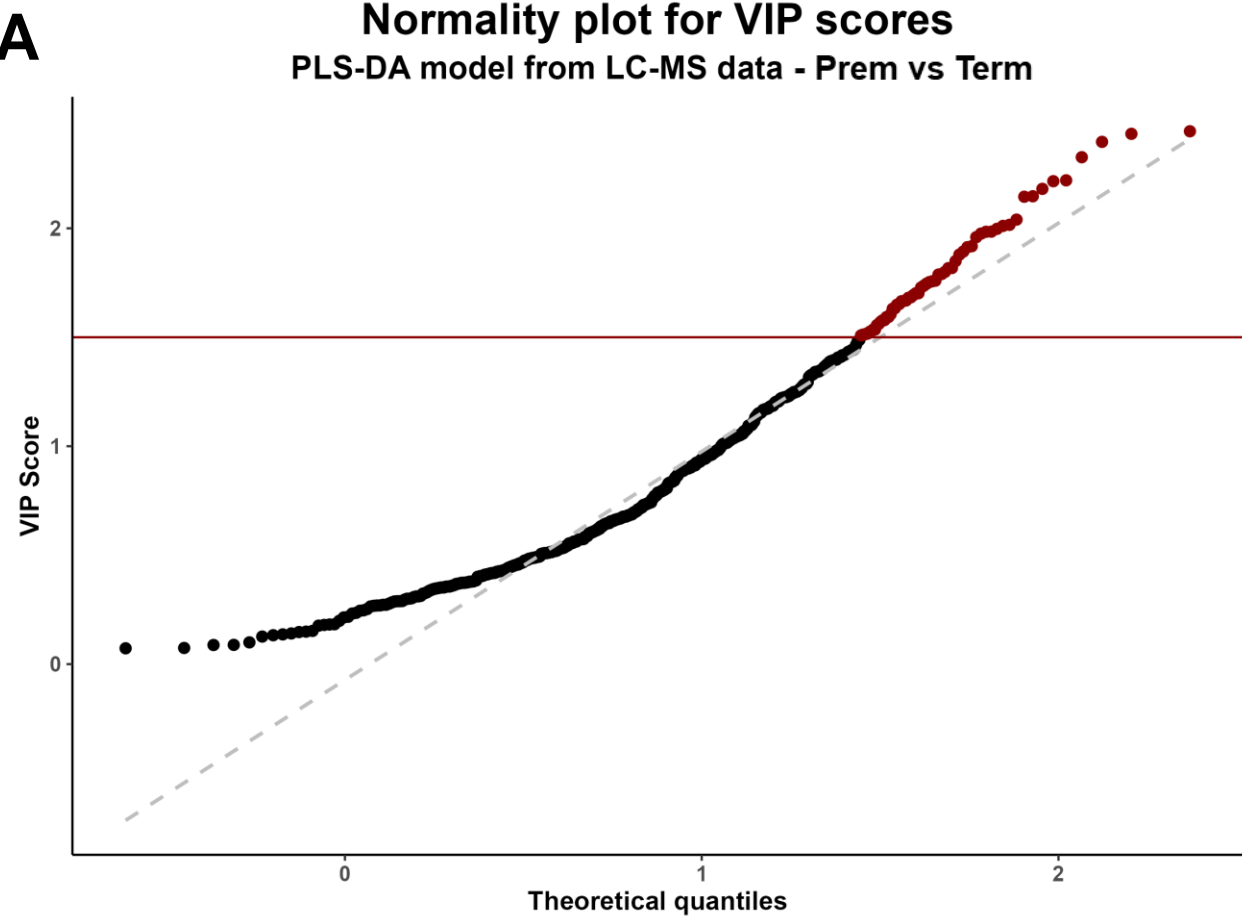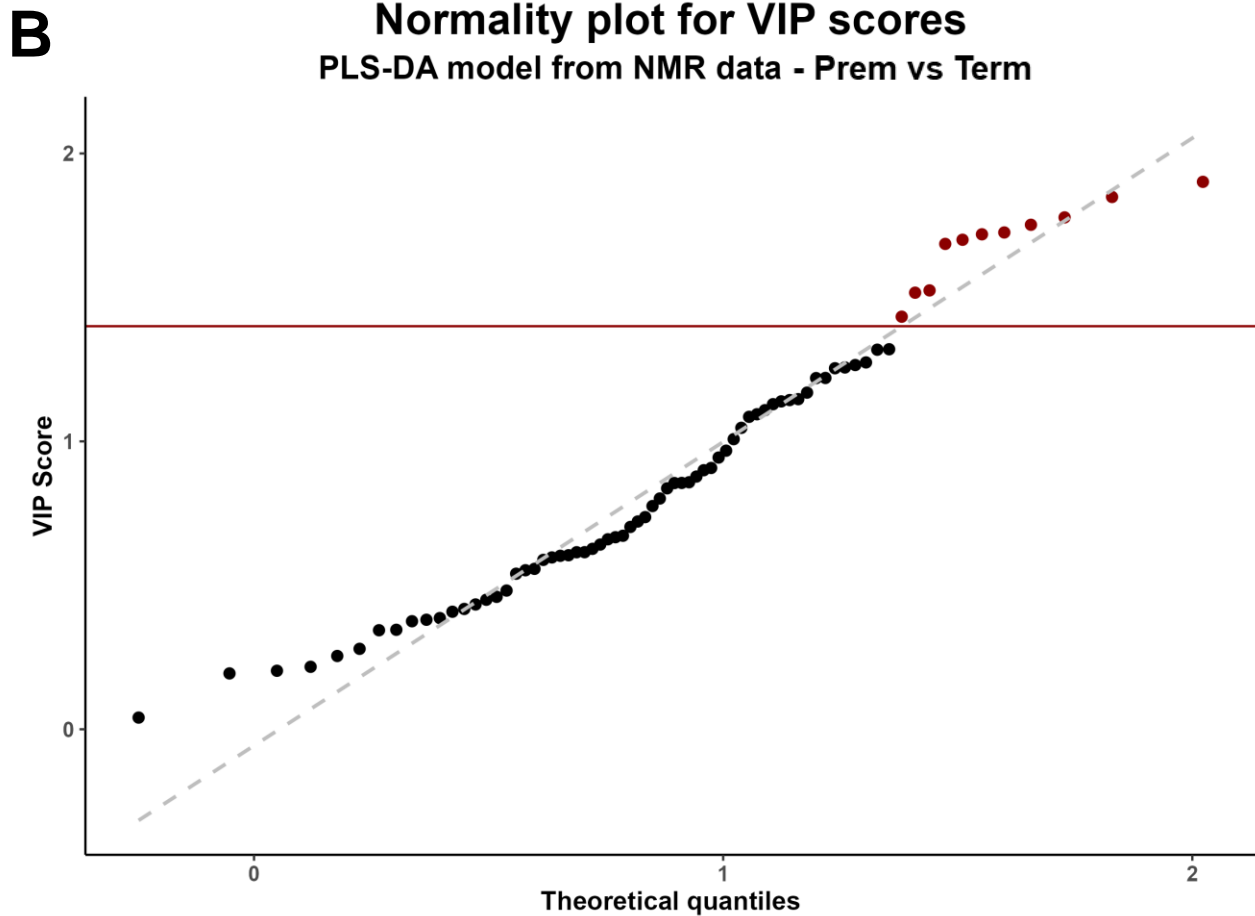

Supplement: Supplementary file 1 [file ijms-26-08953-s001.zip › Figure S1.pdf]

Figure S2

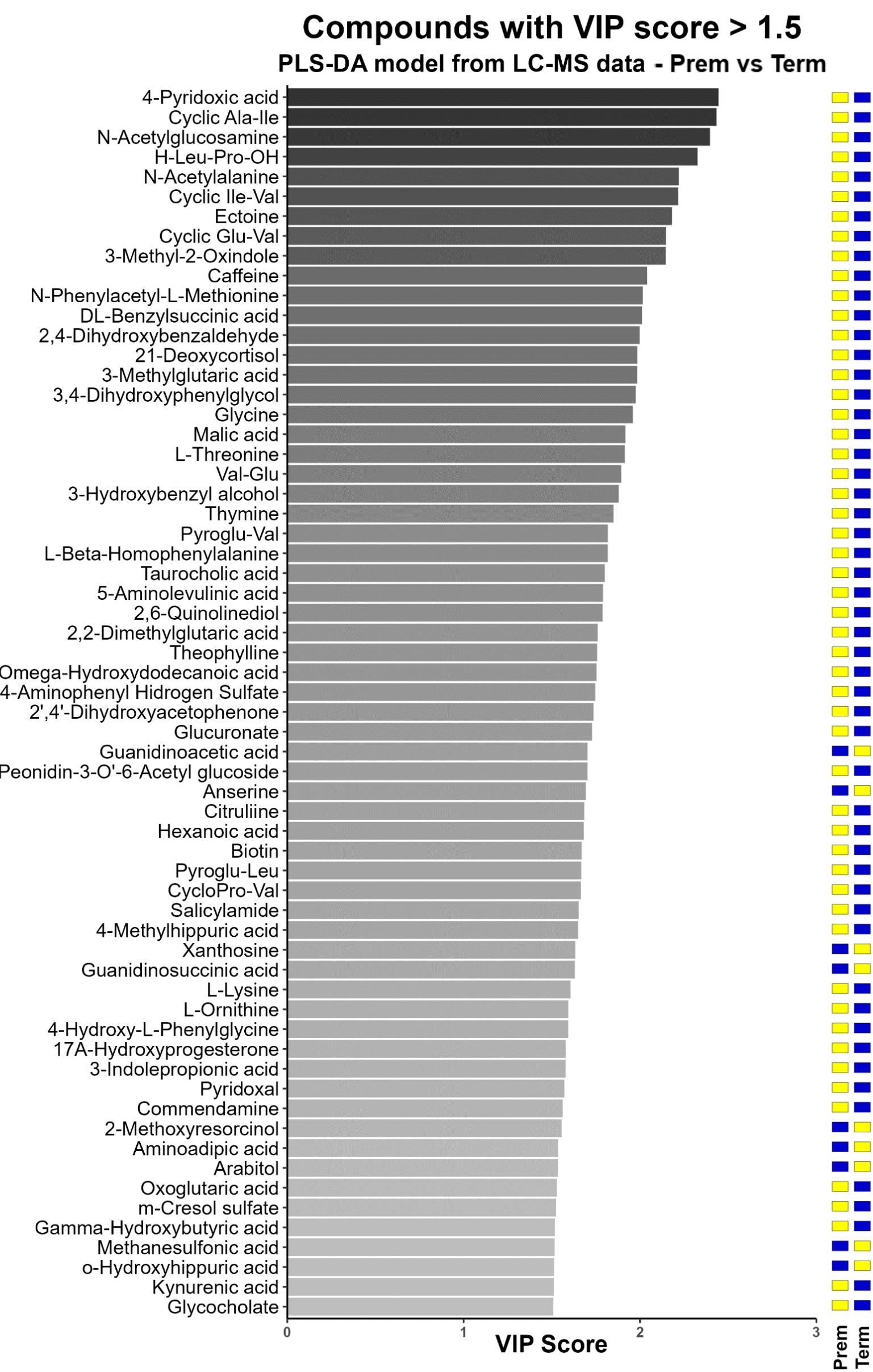

Supplement: Supplementary file 1 [file ijms-26-08953-s001.zip › Figure S2.pdf]

Figure S4

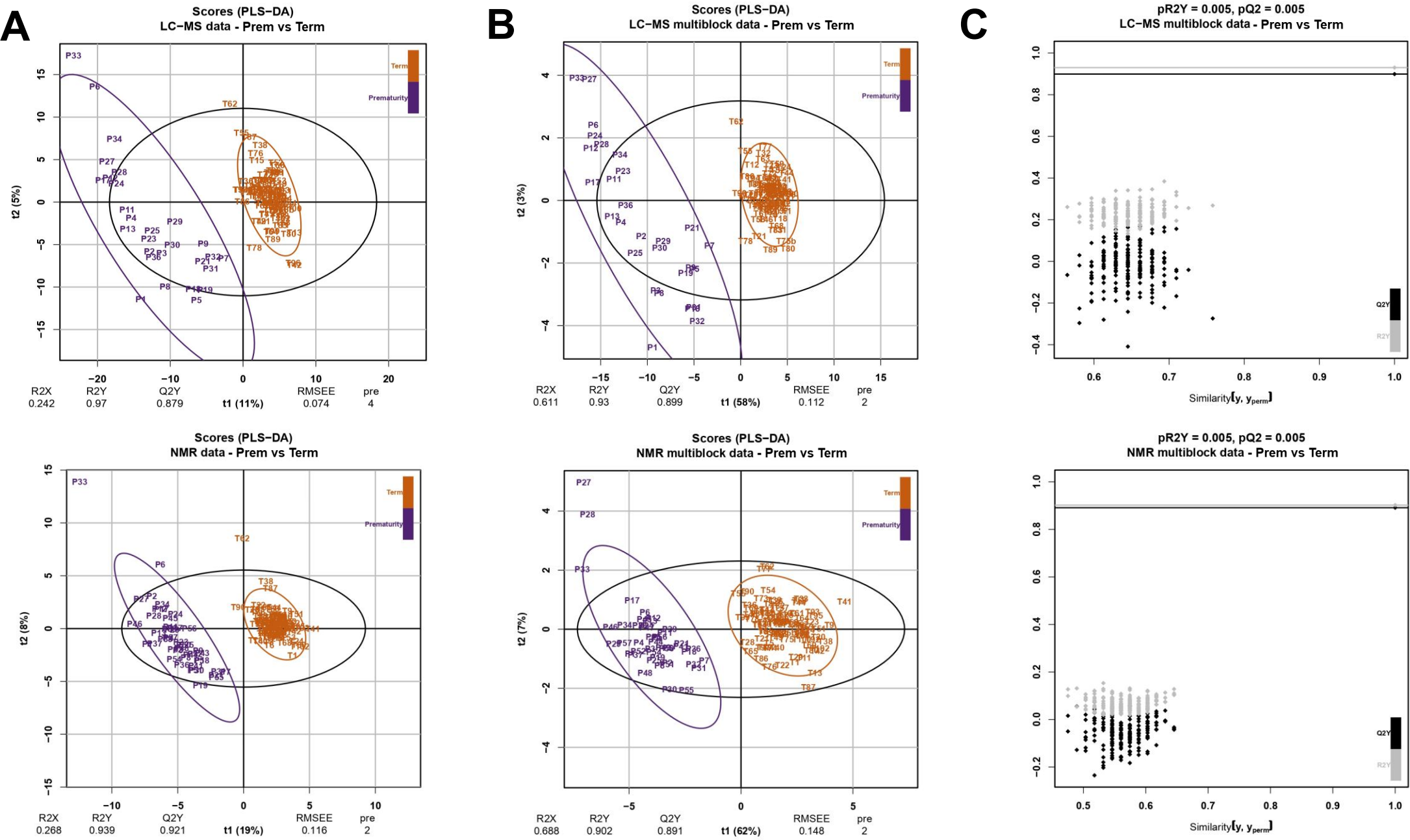

Supplement: Supplementary file 1 [file ijms-26-08953-s001.zip › Figure S4.pdf]
